# Targeted Dealumination via In Situ Activation of Persulfate in Size-Selective Zeolite Channels

**DOI:** 10.1021/acs.jpclett.5c00955

**Published:** 2025-06-05

**Authors:** Youdong Xing, Guangchao Li, Yi Zhang, Jochi Tseng, Dong Fan, Tianqi Cheng, Yung-Kang Peng, Tsz Woon Benedict Lo, Keizo Nakagawa, Shik Chi Edman Tsang, Molly Meng-Jung Li

**Affiliations:** † Interdisciplinary Institute of NMR and Molecular Sciences, 47900 Wuhan University of Science and Technology, Wuhan 430081, China; ‡ Hubei Province for Coal Conversion and New Carbon Materials, School of Chemistry and Chemical Engineering, 47900 Wuhan University of Science and Technology, Wuhan 430081, China; § Department of Applied Physics, 26680 The Hong Kong Polytechnic University, Hong Kong 999077, China; ∥ Department of Applied Biology and Chemical Technology, 26680 The Hong Kong Polytechnic University, Hong Kong 999077, China; ⊥ Diffraction and Scattering Division, 133704 Japan Synchrotron Radiation Research Institute, Spring-8, Sayo-gun, Hyogo 679-5198, Japan; # National Engineering Research Center of Lower-Carbon Catalysis Technology, Dalian National Laboratory for Clean Energy, Dalian Institute of Chemical Physics, Chinese Academy of Sciences, Dalian 116023, China; g Department of Chemistry, 53025 City University of Hong Kong, Hong Kong 999077, China; h Research Center for Membrane and Film Technology, Graduate School of Science, Technology, and Innovation, Kobe University, 1-1 Rokkodai, Nada, Kobe 657-8501, Japan; i Wolfson Catalysis Centre, Department of Chemistry, 6396 University of Oxford, Oxford OX1 3QR, U.K.; j Shenzhen Research Institute, 26680 The Hong Kong Polytechnic University, Shenzhen, Guangdong 518057, China

## Abstract

Selective modification of zeolite structure at specific sites is crucial for optimizing catalytic performance. Tailoring the framework aluminum (Al) siting within particular channels is highly desired but remains challenging. Here, we introduce a persulfate-based dealumination strategy that enables selective removal of Al atoms through in situ activation and zeolite size-selective features. By delivering persulfate molecules into the 12-ring channels of mordenite zeolite, the method employs mild thermal treatment to activate these molecules, releasing etching species that preferentially remove Al from the 12-ring channels while preserving Al sites in the 8-ring channels. Such selective dealumination enhances catalyst longevity while maintaining high catalytic activity in the dimethyl ether carbonylation reaction. The strategy’s effectiveness is confirmed through a combination of advanced characterization techniques, including in situ synchrotron X-ray diffraction, in situ high-energy X-ray total scattering, and probe-assisted solid-state nuclear magnetic resonance. This pioneering approach opens new opportunities for designing tailor-made zeolite structures for advanced catalytic applications.

## Graphical abstract

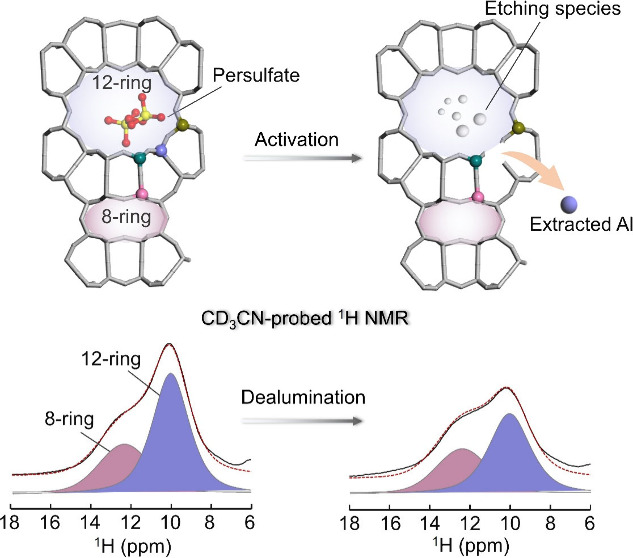

Site-selective structural modification is of great importance for zeolite rational design.^1,2^ The precise placement or removal of active sites within the specific channels and cavities of zeolites has a significant impact on the effectiveness and durability of zeolite adsorbents and catalysts.^3^ This influence arises from the distinct geometric characteristics of these sites and the corresponding confinement effects.^2^ For example, metal sites that are preferentially located within the confined 10-ring channels of **MFI**-type ZSM-5 zeolite demonstrate enhanced stability when exposed to harsh temperature conditions.^3^ In addition, sodium cations residing in the 8-ring channels of mordenite (**MOR**) zeolites show promise for the carbon dioxide capture process even at low concentrations.^4^ Moreover, the Brønsted protons in the constricted 8-ring environment of **MOR** are widely recognized as favorable catalytic sites for various important reactions, including alkane cracking,^5^ syngas conversion,^6,7^ and carbonylation reactions of dimethyl ether (DME) to methyl acetate (MA).^8–10^

The presence of these functional active centers (such as protons, metal, and metal-oxo cations) within specific cavities or channels in zeolites can be achieved through the precise crystallographic placement of negatively charged framework atom tetrahedra (T), typically aluminum (Al) tetrahedra.^11–14^ Hence, for both fundamental understanding and practical considerations, customizing the placement of framework Al within specific channels is highly desired to influence the performance of zeolites. To date, significant efforts have been dedicated to achieving precise control over the framework Al atom placement in zeolite through direct synthetic methods.^15,16^ On the other hand, postsynthetic methods for precise modification are relatively uncommon. Although postsynthetic dealumination is widely employed in industrial applications due to its environmental friendliness, flexibility, and scalability, achieving selective removal of Al in specific locations remains a challenging endeavor.^17–20^ This challenge primarily arises from the uncontrollability of the dealumination process, which results in the random removal of Al atoms and significant disruption of the local framework due to harsh conditions often required for dealumination, such as nitric acid or alkaline solutions, and high-temperature steaming.^9,21^

Recently, there has been increasing interest in exploiting the intrinsic channel-size selectivity of zeolites on the modifiers for the postmodification of specific Al sites or their corresponding Brønsted acid sites (BAS).^9,22^ These approaches utilize size-specific modifiers that can be exclusively introduced into the selective zeolite channels to access and alter undesired acidic sites.^23^ However, the diffusion of the few identified workable modifiers (such as SiCl_4_) within the zeolite channels is severely restricted, resulting in inefficient Al removal, with dealumination occurring only within the nanometer-range from the outer surface of zeolite crystal particles.^9,24^

The above-mentioned synthetic challenges and limitations prompt us to develop novel methodologies or treatments for selective dealumination. In this study, we propose integrating a novel *in situ* etching technique with the fundamental size-selective strategy in zeolite to achieve targeted dealumination. Our method innovatively utilizes thermally activable persulfate to release effective dealumination species *in situ* within the targeted zeolite channel, which was previously unattained in the field. As depicted in Figure 1, NH_4_-form **MOR** zeolite was selected as an example to demonstrate the persulfate-based dealumination strategy. **MOR** zeolite is a frequently used zeolite known for its distinctive structure comprising 12-ring and 8-ring channels connected by 8-ring side pockets.^8^ The Al residing in the T3 site of the 8-ring channels is often recognized as the most favorable for catalytic activity compared to other Al sites (T1, T2, and T4) in the 12-ring channel.^4–10^ In this work, ammonia persulfate (APS), a commonly used chemical in industrial metal dissolution,^25^ is employed as the dealumination agent. APS generally exhibits temperature-dependent activation behavior, remaining inert at low temperatures but decomposing into reactive species upon thermal stimulation.^26,27^ Persulfate molecules have an estimated size of 5.7 Å,^28^ allowing them to enter the 12-ring windows (6.5 × 7.0 Å) of **MOR** but restricting them from accessing its narrower channels through the 8-ring windows (2.6 × 5.7 Å) and the 8-ring side pockets (4.8 × 3.4 Å).^8^ By leveraging the pore-size selectivity of MOR and the thermal sensitivity of APS, we can selectively introduce APS molecules into the 12-ring channels of the MOR zeolite at low temperatures, ensuring their inactivity during this stage, as demonstrated in the subsequent discussion.

**Figure 1:**
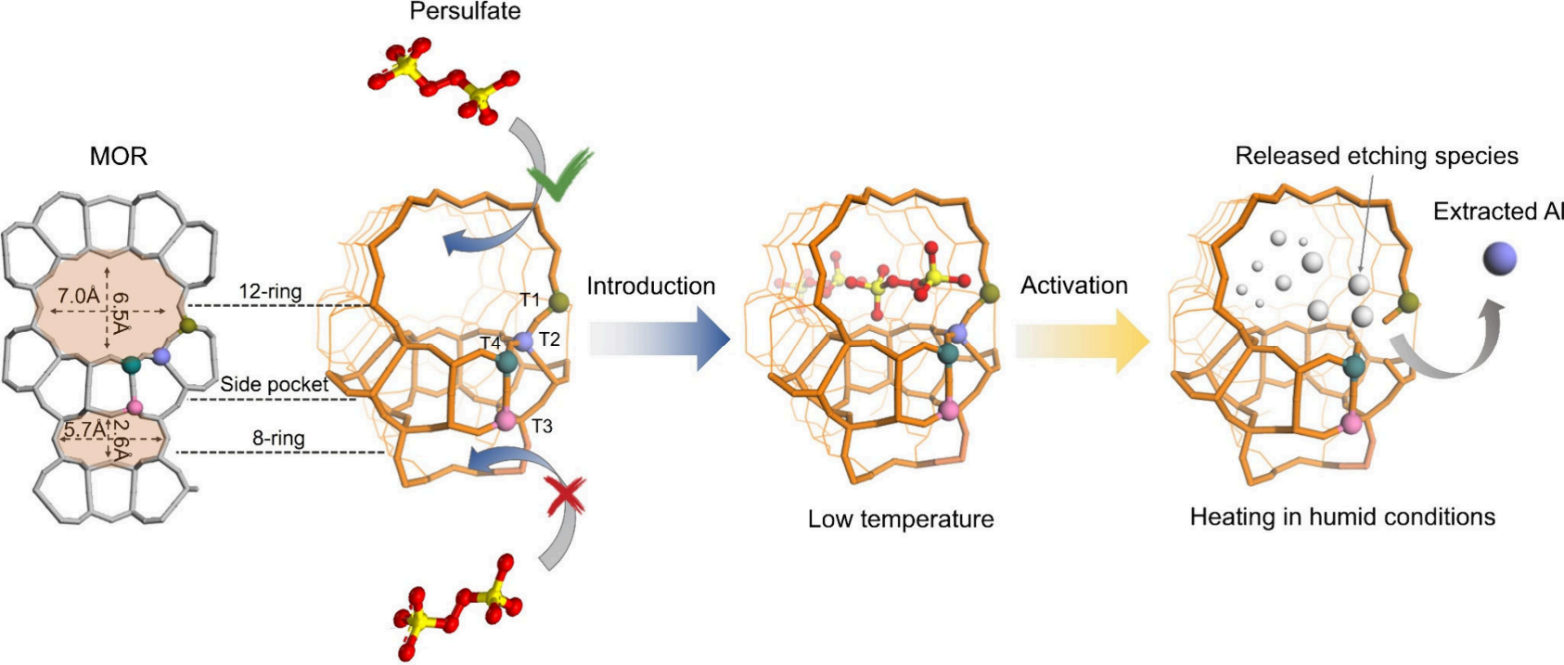
Process involving targeted delivery of persulfate molecules into specific 12-ring channels, followed by thermal activation to release etching species and extract Al atoms from the 12-ring channels while leaving the 8-ring channels completely unaffected. An example illustration demonstrates the removal of the T2 Al atom from the 12-ring site.

Subsequently, APS in **MOR** could be activated by heating, leading to *in situ* decomposition in the 12-ring channels and the release of etching species, such as radicals and protonic acids (see eqs 1 and 2).^29,30^ The protonic species are recognized for their role in hydrolyzing the framework Al–O bonds for dealumination.^17^ Furthermore, •OH radicals might contribute to Al removal, given their ability to promote zeolite synthesis by facilitating the breaking and formation of framework bonds.^31^ However, distinguishing the degree of contribution of each species in the dealumination process is challenging, as they are generated simultaneously during the persulfate decomposition process (eq 2). Upon activation, due to a preferential siting of persulfate molecules in **MOR**, these *in situ* formed etching species are expected to exclusively dealuminate T1, T2 and T4 sites in the 12-ring channels while preserving T3 Al sites in the 8-ring channels.

1 S2O82−→2S•O4−

2 S•O4−+H2O→H++SO42−+O•H

The integration of advanced characterization techniques forms the methodological foundation of this study, critically enabling atomic-scale resolution of selective dealumination processes. Recognizing the complexity of multistep interactions between zeolite and persulfate—where persulfate activation, radical/acid generation, and framework modification occur synergistically within zeolite channels—we strategically combine multiple complementary approaches: (1) Synchrotron X-ray diffraction (SXRD) precisely resolves APS distribution within MOR zeolite channels and tracks temperature-dependent interaction between APS and zeolite framework during thermal activation, (2) High-energy X-ray total scattering (HEXTS) maps disrupted or distorted framework with subnanometer resolution, and (3) Probe-assisted solid-state NMR (SSNMR) distinguishes Al coordination environments with site specificity. This comprehensive analytical complementarity establishes a direct correlation between preferential persulfate activation in 12-ring channels and resolves dealumination selectivity, advancing molecular-scale mechanistic understanding of our persulfate-based dealumination strategy.

To determine the optimal temperature for introducing and activating APS in **MOR** zeolite, we employed UV–vis spectroscopy to track the change in APS concentration over time at various temperatures, including room temperature (RT), 40 °C, 80 and 120 °C. This method is commonly employed to detect APS activation or decomposition.^32^ Figure 2a illustrates the time-dependent change in relative APS concentration (C/C_0_) at each temperature, with analysis details shown in Figure S1. It is observed that the APS concentration in the APS/MOR mixture remains constant at RT for 24 h. However, when exposed to the elevated temperatures (40 °C, 80 °C, and 120 °C), the concentration of APS exhibits a decrease that becomes more significant with higher temperatures. The observation clearly indicates that the decomposition/activation process of APS is temperature-driven, and 80 °C is selected for the activation of APS in this study.

**Figure 2:**
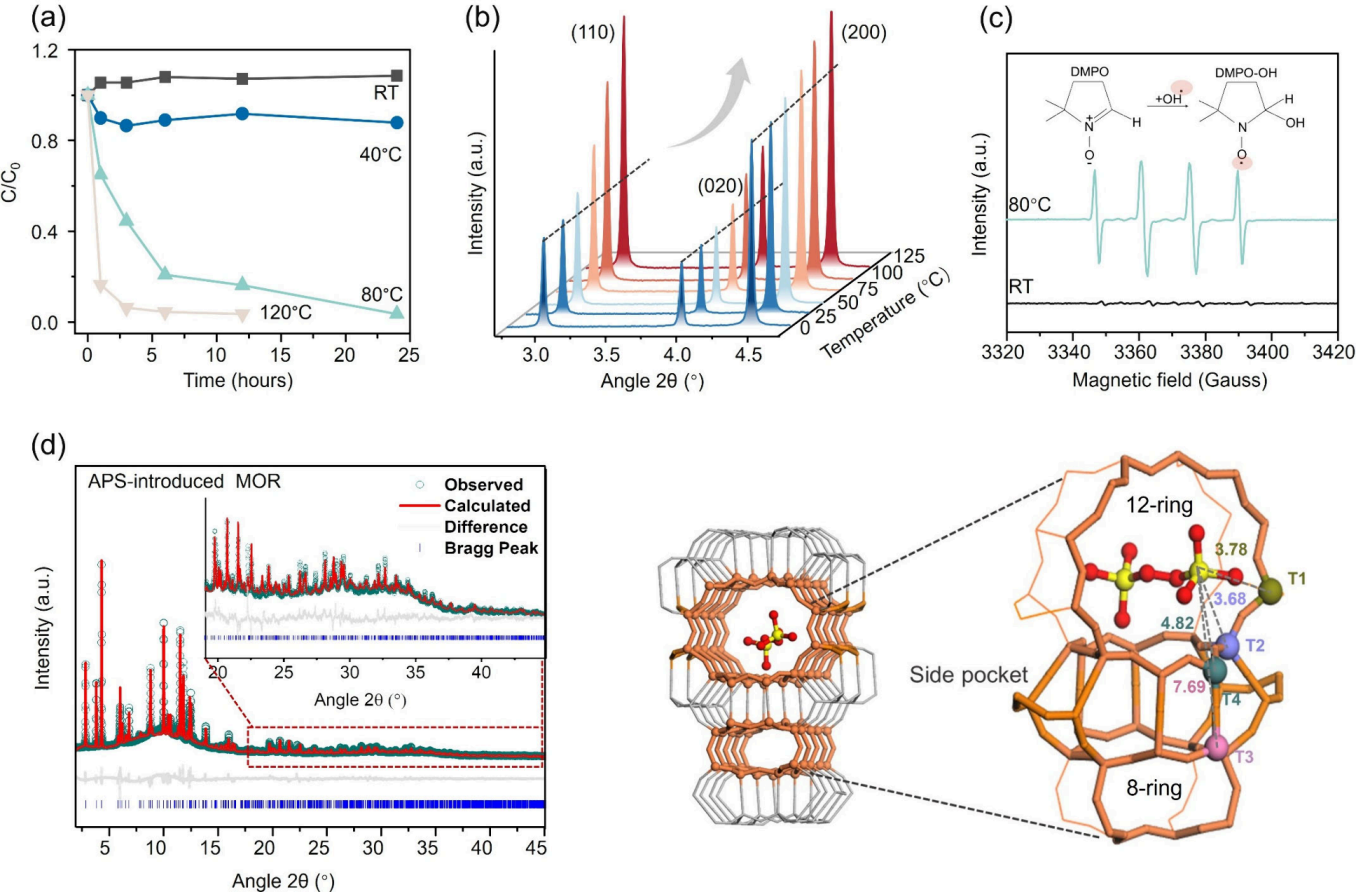
(a) Change in the relative APS concentration (C/C_0_) over time at various heating temperatures obtained from the UV–vis absorption spectra. Analysis details are listed in Figure S1. (b) Temperature-dependent SXRD data of the APS-introduced **MOR** zeolite from 0 to 125 °C. (c) EPR spectra of the APS-introduced **MOR** zeolite treated at RT and 80 °C. (d) SXRD patterns and Rietveld refinement profiles of the APS-introduced **MOR** zeolite. SXRD data in the range of 19–45° are expanded to illustrate the quality of the Rietveld refinement. Crystallographic model based on SXRD Rietveld refinement and the interatomic distance between the S of the persulfate and the atoms in different T sites. Symmetry in adsorption sites is disregarded for the sake of clarity.

We further investigated the changes in the **MOR** framework structure and the interactions from APS during thermal activation through a temperature-dependent high-resolution SXRD experiment. In Figure S2, the full SXRD profiles (2θ = 2–80°) of the APS-introduced **MOR** zeolite show minimal changes in **MOR** structure within the low-temperature range (0 to 25 °C). However, upon heating, a gradual enhancement of small-angle peaks (2θ < 6°) emerges (Figure 2b), coinciding with the thermal decomposition of APS within the 12-ring channels as independently confirmed by UV–vis spectroscopy (Figures 2 **a and** S1). During heating, bulk APS decomposes into smaller fragments (e.g., sulfates and radicals) with higher mobility. These fragments may partially expel from the channels or redistribute within them, reducing disordered guest-framework interactions. This process likely allows the **MOR** framework to relax toward its intrinsic ordered configuration, thereby enhancing the low-angle XRD signals, which are sensitive to the long-range periodicity of the framework. The observed trend aligns with prior studies^33,34^ where guest removal or redistribution during heating intensifies low-angle SXRD signals by minimizing scattering interference from disordered adsorbates. These SXRD trends, supported by complementary UV–vis data, underscore the critical role of temperature in triggering APS decomposition, which is a crucial aspect of our targeted dealumination strategy.

Therefore, in our dealumination approach, we first introduce APS into the zeolite channels at RT, and then proceed with a mild and controllable activation of APS at 80 °C. Electron paramagnetic resonance (EPR) spectroscopy using 5,5-Dimethyl-1-pyrroline N-oxide (DMPO) as a spin-trapping agent revealed radical formation. Control experiments conducted on untreated MOR zeolites at RT and 80 °C exhibit no radical signals (Figure S3 **)**, confirming that the zeolite has no intrinsic activity under these conditions. As shown in Figure 2c, the APS-introduced **MOR** zeolite at RT does not show any radical signals. In contrast, the APS-introduced **MOR** zeolite heated at 80 °C exhibits a quartet pattern with a peak integral of 1:2:2:1, indicating the presence of the DMPO–•OH adduct. Note that the DMPO–•SO_4_ signal is not observed, likely due to DMPO’s higher affinity for •OH compared to •SO_4_^–^.^35^ These observations confirm that APS retains inactive in **MOR** at RT, but undergoes activation at 80 °C, resulting in the *in situ* release of reactive species that are capable of removing Al from the structure (eqs 1 and 2).

Electron microscopy examination of the vacuum-dried APS-introduced **MOR** sample shows a uniform distribution of S elements within the crystals, confirming the homogeneous penetration of persulfate molecules into the zeolite (Figure S4). To inspect the configurations and residence of the introduced APS molecules within **MOR**, high-resolution SXRD was employed. SXRD is a well-established technique renowned for its precision in determining crystallographic positions and has more recently been extended to probe adsorbates within porous crystalline materials such as zeolites and metal–organic frameworks (MOFs).^36,37^ In this study, SXRD measurements were conducted at −173 °C to minimize the thermal motion of APS molecules and reduce structural distortion to improve precise crystallographic analysis.^38^ Crystal structures of the collected diffraction data were carefully refined using the full-pattern Rietveld refinement method, which could elucidate the spatial relationships between guest APS species and the host zeolite frameworks, providing precise interatomic distances and angles with acceptable experimental error.

The SXRD patterns for both the pristine **MOR** and the APS-introduced **MOR** zeolite are presented in Figure S5. The difference in their Bragg’s peak intensities is significant, resulting from the introduction of guest persulfate into the zeolite. The well-refined diffraction data of both samples, with the small discrepancies in the gray difference profiles, are shown in Figures 2 **d and** S6, and the detailed structural parameters are summarized in Tables S1–S4 and Figure S5. The refined structure of the APS-introduced **MOR** (Figure 2d) illustrates the interatomic distances between the introduced APS molecules and the framework atoms located at different T sites. Notably, the S atom of the APS molecule exhibits the closest distance (3.68 Å) to the atom in the T2 site, followed by T1 (3.78 Å) and T4 (4.82 Å) in the 12-ring channel. The longest distance (7.69 Å) is observed between S and T3 in the 8-ring channel. This observation suggests that the introduced APS molecule exclusively resides in the 12-ring channel and is oriented parallel to the channel, facing the 12-ring window (Figure 2d). While the specific APS-T-site distances measured at −173 °C may vary dynamically at elevated temperatures due to thermal effects, the inherent steric bulk of APS molecules prevents their penetration into the narrower 8-ring channels under the experimental conditions.

The crystallographic analysis has confirmed the selective residence of persulfate molecules within the zeolite channel at an atomic level, offering insights into the spatial coordinates of the introduced APS molecules in relation to specific framework sites. This information is particularly significant as it validates the channel-size selectivity of **MOR** toward APS molecules, together with the previously confirmed *in situ* activation of APS in **MOR**, collectively validating the foundation for our persulfate-based dealumination strategy.

The **MOR** zeolites introduced with APS were then subjected to treatment at 80 °C for 24 h under a humidified N_2_ atmosphere (200 mL/min flow rate, saturated with water vapor at 25 °C), yielding the APS-activated sample designated as MOR-APS-80C. The indispensable role of water vapor in enabling APS-mediated dealumination was confirmed by control experiments under dry N_2_ flow, where no framework Al removal occurred in the absence of moisture (Figure S7). This observation aligns with our mechanistic hypothesis wherein water facilitates the generation of reactive species (•OH radicals and H^+^ ions) via sulfate radical hydrolysis. Following activation, all samples were extensively washed with deionized water and subsequent drying to eliminate any remaining species within the zeolite channels. Elemental analysis confirmed sulfur contents below 0.1 wt % in both pristine and APS-treated **MOR** samples, demonstrating the effective elimination of residual sulfur species via washing with deionized water after APS treatment, as detailed in Experimental Section in SI. For comparison, the **MOR** sample introduced with APS but not subjected to elevated temperature treatment is referred to as MOR-APS-RT.

Structural analysis reveals that all APS-treated samples maintain a distinct **MOR** structure with high crystallinity and exhibit a porous texture similar to that of the pristine **MOR** (Figure S8). Additionally, scanning electron microscopy (SEM) images (Figure S9) show no obvious changes in the surface and morphology of **MOR** before and after APS treatment. These findings suggest that the APS treatment does not cause macroscopic structural damage to the **MOR** zeolite, preserving its structural integrity.

To reveal the influence of the atomic-level local structure of **MOR** zeolite resulting from APS activation at 80 °C, we conducted *in situ* HEXTS measurements (see Supporting Information for HEXTS experiment details). The pair distribution function (PDF), G­(r), obtained from the measurements allows to identify various atomic distances associated with framework connectivity.^39^ In Figure 3a, specific peaks are seen at distances of 1.7, 2.7, and 3.2 Å, which correspond to the correlation between T-O, O–O, and T-T atom pairs, respectively.^40^ The distances in the range of 3.6 to 4.7 Å provide valuable insights into the ring structures within the zeolites. From the analysis, the signal corresponding to 4-ring or 5-ring (4/5-rings) is observed at a distance of 4.1 Å, while the signal for the 6-ring or larger (≥6-rings) is observed at distances of 4.4 Å.^18,40^ With increasing activation time, the well-separated signals associated with the 4/5-ring and ≥ 6-ring merge into a broader peak, accompanied by a decrease in peak intensity. This observation reflects the fact that the building ring units within the **MOR** structure are disrupted or distorted in the elevated temperature, suggesting the alteration in the coordination of framework atoms by *in situ* formed etching species during APS activation.^18^

**Figure 3:**
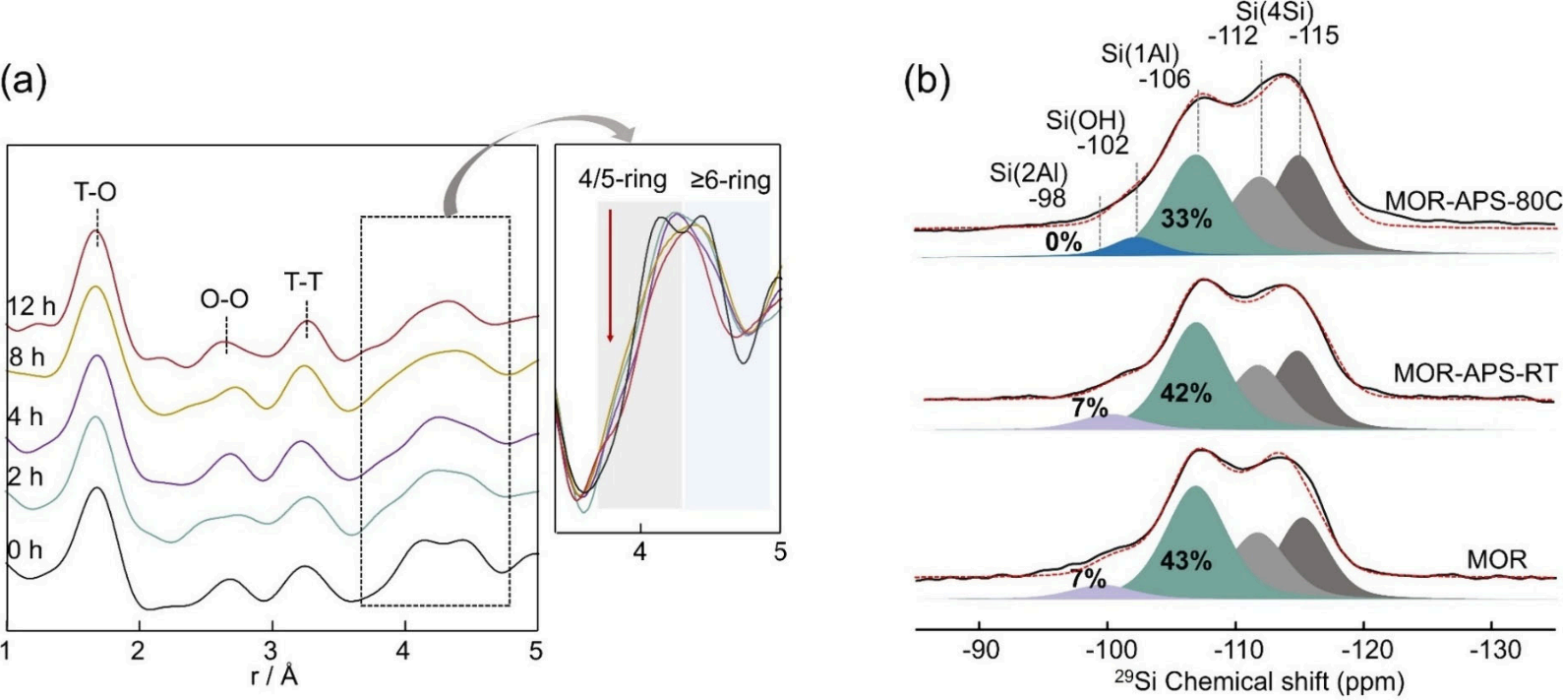
(a) Reduced pair distribution function *G*(*r*) of the APS-introduced MOR treated at 80 °C for 0–12 h. The region of ring-related signals is enlarged. (b) Weight-normalized ^29^Si single-pulse MAS SSNMR spectra of as-made samples. The percentages of the peak areas are relative to the total integral area of the pristine **MOR** zeolite.

The dealumination behavior of APS-treated MOR zeolites was comprehensively analyzed using SSNMR spectroscopy. As shown in the ^29^Si single-pulse MAS SSNMR spectra (Figure 3b), four distinct resonance signals are observed at δ = – 112 to – 115 ppm (Si­(4Si)), – 106 ppm (Si­(1Al)), – 102 ppm (Si–OH), and – 98 ppm (Si­(2Al)), corresponding to different silicon coordination environments in the zeolite framework.^9^ Notably, MOR-APS-RT exhibits patterns similar to those of the pristine **MOR**, indicating that there is no significant change in these framework species during the APS introduction step, affirming the inertness of inactivated APS in the zeolite framework. It is evident that framework Al removal occurs in the thermal treatment at 80 °C, as observed in the MOR-APS-80C sample. The Si­(1Al) resonance signal experiences a significant decrease over the activation period, accompanied by the disappearance of the Si­(2Al) peak (Figure 3b). These results unequivocally demonstrate that thermally activated APS serves as an effective dealumination agent, capable of removing Al atoms from the zeolite framework. Note that the contribution of heating to the removal of framework Al is excluded by subjecting the pristine **MOR** zeolite to treatment at 80 °C (Figure S10).

Upon examining the spatial chemical composition of the pristine and APS-treated zeolites via XRF, NMR, and XPS analysis, it is observed that the framework and surface Si/Al ratios remain consistent (refer to Table 1). This signifies the extensive and homogeneous penetration of APS and the effectiveness of our dealumination strategy throughout the entire zeolite crystals. This uniform dealumination is attributed to the unique *in situ* release of etching species in our strategy, which overcomes any potential hindrance caused by diffusion limitations. Note that the consistency between bulk and framework Si/Al ratios unambiguously confirms the effective elimination of extra-framework Al species during the washing step after dealumination (as detailed in the Supporting Information, Experimental Section).

**Table 1:** Composition Analysis and Distribution of Framework Al Atoms and BAS in the Pristine and APS-Treated **MOR** Samples

				Al atom location				
	spatial Al distribution			framework Al^d^ (%)			BAS^e^ (μmol/g)	
sample	bulk Si/Al^a^	framework Si/Al^b^	surface Si/Al^c^	8-ring	12-ring	dealuminated	8-ring	12-ring
MOR	7.72	7.12	7.30	28	72	0	538	1391
MOR-APS-RT	7.68	7.09	7.85	28	72	0	536	1394
MOR-APS-80C	10.20	10.06	10.98	27	48	25	521	967

a Determined by XRF.

b Determined with ^29^Si MAS SSNMR data by the equation ∑_*n*=0_^4^ *I*_Si(OAl)_*n*__/0.25∑_*n*=0_^4^ *nSI*_Si(OAl)_*n*__.

c Determined by XPS analysis.

d Data determined from the weight-normalized ^27^Al NMR spectra. The percentages are evaluated from the individual ^27^Al NMR peak areas in the pristine and APS-treated **MOR** zeolites relative to the total peak area of the pristine **MOR** zeolite.

e Determined with CD_3_CN probe-assisted ^1^H MAS SSNMR data by the calibration line of standard samples (see Figure S11).

Detailed insights into the specific location of framework Al after APS dealumination were comprehensively elucidated in a series of one-dimensional (1D) and two-dimensional (2D) NMR experiments. Notably, all ^27^Al NMR analyses were performed on hydrated MOR samples prepared by exposing the materials to ambient air for 3 days to ensure complete hydration. Hydration is essential for ^27^Al NMR characterization as it symmetrizes the local electronic environments of framework Al sites, suppressing quadrupolar interactions and enabling clearer resolution of distinct Al coordination states.^41^ In the 1D ^27^Al MAS NMR experiment (Figure 4a), a tetrahedral framework Al signal at 55 ppm is observed in all the samples,^10^ and a 0-ppm signal appears in the MOR-APS-80C sample due to the presence of a small quantity of residual extra-framework Al species.^42^ Analysis of the enlarged region of the framework Al signal in Figure 4a reveals a decreased peak area in MOR-APS-80C, accompanied by an upfield shift, suggesting a change in framework Al distribution. It is noteworthy that no significant change in framework Al concentration is observed during the APS introduction step, as evident from the similar patterns between **MOR** and MOR-APS-RT. This reaffirms the inertness of APS to the zeolite framework at room temperature, which is consistent with the ^29^Si NMR results (Figure 3b).

**Figure 4:**
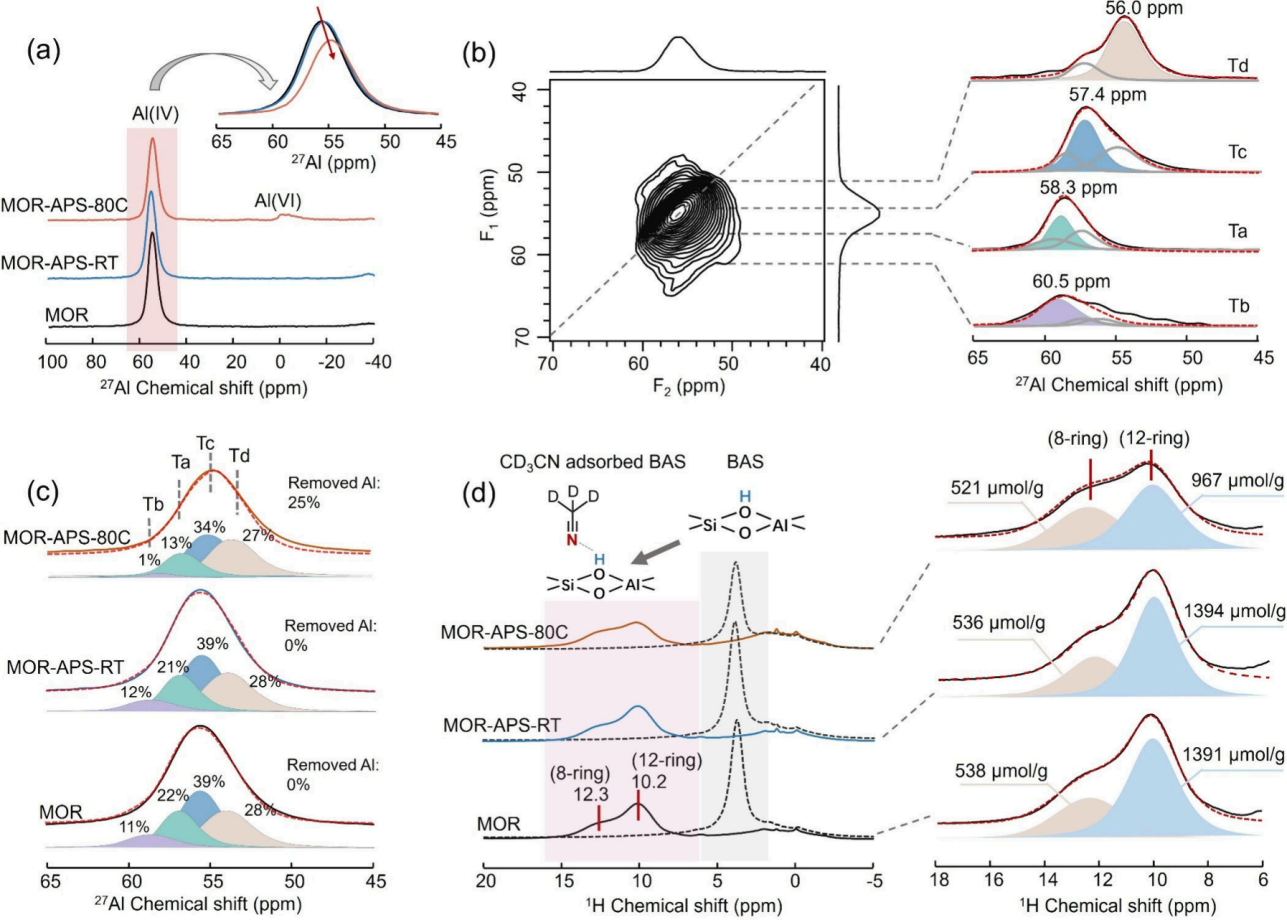
(a) Weight-normalized 1D ^27^Al MAS SSNMR spectra at 11.6 T and the enlarged region for the framework Al signal of the pristine **MOR** and APS-treated **MOR** samples. (b) ^27^Al MQ MAS SSNMR spectrum of pristine **MOR** at 11.6 T and slices extracted from the F1 dimension. (c) Deconvoluted ^27^Al MAS SSNMR spectra of the as-made samples. Note that the percentages of the peak areas are relative to the total integral area of the pristine **MOR** zeolite. (d) ^1^H MAS SSNMR spectra of the samples before (dotted line) and after CD_3_CN adsorption (solid line). The enlarged region for the deconvoluted BAS-related signals of the CD_3_CN-adsorbed samples. Red dashed lines represent simulated spectral lines.

To better distinguish different Al T-sites, we conducted 2D ^27^Al multiple-quantum (MQ) MAS NMR as this technique alleviates the quadrupolar effect of ^27^Al. Figure 4b shows the MQ spectra of the pristine **MOR** and the extracted representative slices in isotropic F1 dimension, which demonstrates the distinguished Al environment by different chemical shifts. The MQ spectrum of the APS-thermally treated **MOR** sample is supplemented in Figure S12, which shows a similar Al environment. The isotropic chemical shifts (δ_iso_) and quadrupole interaction constants (C_Q_), obtained from the fitted slices, are summarized in Table S5. Using these quadrupolar parameters, 1D MAS spectra were deconvoluted and simulated into four components, around 58.3, 60.5, 57.4, and 56.0 ppm, assigned to Al at Ta, Tb, Tc, and Td sites, respectively. Quantitative analysis of the deconvoluted peak areas (Figure 4c and Table 1) demonstrates that APS thermal treatment induces site-selective dealumination: the Td site proportion remains nearly constant (28% pre- and post-treatment), while the combined Ta–Tc contribution drops from 72% to 48%.

This selective behavior is further corroborated by acid site distribution analysis using deuterated acetonitrile (CD_3_CN) as an NMR probe. CD_3_CN’s small kinetic diameter (∼0.38 nm) enables access to BAS in both 8-ring and 12-ring channels, providing precise spatial resolution of acidity.^43^ Prior to adsorption, all samples exhibit a single BAS signal at 4.0 ppm in ^1^H MAS NMR (Figure 4d). Upon CD_3_CN adsorption, this signal splits into two distinct peaks at 12.3 ppm (8-ring T3 sites) and 10.2 ppm (12-ring T1/T2/T4 sites),^10,43^ enabling quantitative tracking of framework Al distribution, shown in Figure 4d and Table 1. The pristine MOR contains 1929 μmol/g framework Al, with 28% (538 μmol/g) in 8-ring T3 sites and 72% (1391 μmol/g) in 12-ring sites. After the thermally activated APS treatment, the 12-ring Al content decreases sharply to 967 μmol/g, while the 8-ring Al (T3 sites) remains nearly constant throughout the APS treatment. Note that the quantitative correlation between CD_3_CN-probed acidity and ^27^Al NMR deconvolution allows unambiguous assignment: the Td signal (δ≈56.0 ppm, Figure 4c) corresponds to 8-ring T3 sites, while Ta–Tc signals (δ≈57.4–60.5 ppm) represent 12-ring T1/T2/T4 sites—a framework fully consistent with prior assignments.^9^ In stark contrast, conventional high-temperature steaming (Figure S13) induces nonselective Al removal from both channels, underscoring the unique advantage of our method.

The comprehensive SSNMR observations shown above undeniably validate the success of our innovative persulfate-based dealumination approach. By leveraging size selectivity and precise etchant release within zeolites, our method exclusively removes Al in the 12-ring channel while leaving the T3 Al atoms in the 8-ring channel completely unaffected. Collectively, the selective removal of Al from 12-ring channels—while preserving 8-ring Al sites—demonstrates a remarkable breakthrough in zeolite postmodification. To our knowledge, this work represents the first successful integration of persulfate chemistry into zeolite dealumination, achieving unprecedented spatial control under mild conditions. This advancement not only expands the toolbox for zeolite engineering but also opens avenues for tailoring acid site distributions in complex pore architectures.

The tunability of dealumination is paramount for tailoring zeolite acidity and optimizing catalytic performance. While our methodology employs a 2:1 stoichiometric excess of APS relative to BAS to ensure reagent accessibility, the observed limited Al extraction efficiency (∼25%) probably stems from characteristics inherent to the radical-mediated process under mild conditions. APS decomposition generates sulfate radicals S_2_O_8_^2–^ → 2•SO_4_^–^) and subsequent reactive species (•OH, H^+^ via •SO_4_^–^ hydrolysis), yet their ultrashort lifetimes^44^ are expected to limit cumulative framework interactions and overall dealumination efficiency. To achieve tunable Al extraction, we systematically adjusted treatment parameters, including persulfate concentration (1.7–5 wt %), activation time (12–48 h), temperature (80–250 °C), and iterative dealumination cycles (1–3). The results demonstrate the method’s sensitivity to treatment conditions, enabling framework dealumination with Al removal efficiencies in 12-ring channels reaching up to 40%. This indicates considerable potential for further optimization of both efficiency and site selectivity through refined modulation of treatment parameters (Figure S14).

Persulfate-treated MOR zeolites were further evaluated for their performance in the carbonylation of DME to MA, a reaction highly sensitive to the distribution of BAS within the MOR framework.^10^ Figure 5 illustrates DME conversion and MA selectivity as functions of time on stream (TOS). The MOR-APS-RT sample exhibits trends in DME conversion and MA selectivity similar to those of the parent MOR zeolite, reflecting their comparable Al and acid site distributions (Table 1). Notably, DME conversion for MOR-APS-RT and the parent MOR rapidly decreases from 100% to 70% after the first 4 h, accompanied by a marked decline in MA selectivity. This rapid deactivation is primarily attributed to the high concentration of BAS in the 12-ring channels, which account for 72% of the total acid sites (Table 1), thereby facilitating rapid coke formation and subsequent catalyst deactivation.^8–10^

**Figure 5:**
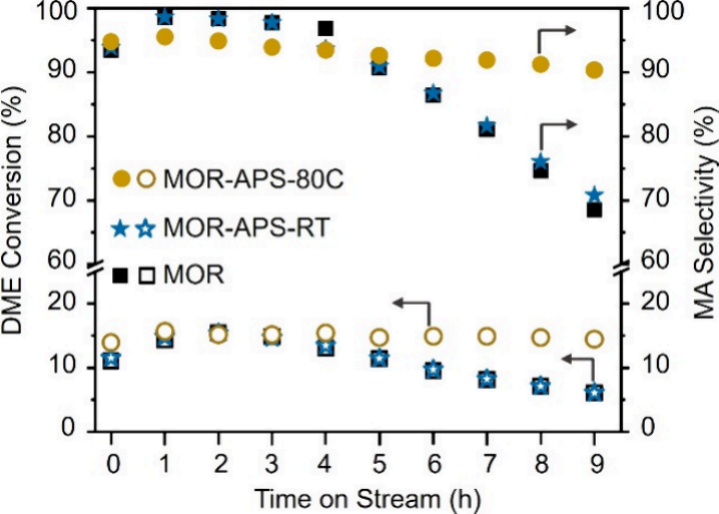
DME conversion and MA selectivity over the parent and persulfate-treated MOR catalysts. Reaction conditions: 200 °C, 2 MPa, 5/35/60 DME/CO/N_2_, GHSV = 3600 mL g^–1^ h^–1^.

In contrast, the MOR-APS-80C sample demonstrates comparable initial catalytic activity but maintains superior long-term stability, with only a slight decline in performance over the test period. This enhanced stability validates the effectiveness of our strategy that the desired catalytic acid sites within the 8-ring channels (specifically T3 Al sites) are preserved during persulfate dealumination, while the acidic sites in the 12-ring channels are selectively removed, effectively mitigating coking.^9^ Notably, another MOR sample subjected to a shorter APS treatment (12 h, achieving 13% Al removal in 12-ring channels) demonstrates a strong correlation between framework Al elimination extent and both stability and MA selectivity when compared to the MOR-APS-80C sample (24-h APS treatment, with 22% Al removal in 12-ring channels), as shown in Figure S15. This comparison further confirms the critical role of 12-ring BAS elimination in performance enhancement for the carbonylation of DME. These catalytic results align with the systematic characterizations of framework Al and acid site distributions, confirming that the proposed strategy effectively optimizes the acidity distribution in MOR zeolites by selective Al removal, thereby improving catalytic performance.

In summary, our pioneering targeted dealumination strategy introduces persulfate postsynthetic treatment into the field of zeolite. Persulfate’s inertness at room temperature and inherent size selectivity in the zeolite framework enables unrestricted diffusion into specific zeolite channels, as convincingly demonstrated by UV–vis, EPR and SXRD observations. Upon mild heating at 80 °C, persulfate becomes activated, leading to precise dealumination in the selected channel, validated by comprehensive PDF and SSNMR analysis. This selective dealumination significantly improves catalyst longevity while preserving high catalytic activity in the dimethyl ether carbonylation reaction. This technique opens up a wide range of possibilities for selectively modifying zeolite materials, enabling us to tailor the positioning of Al atoms within the zeolite framework to optimize catalytic performance. Moreover, the potential for persulfate activation can extend beyond heat and includes other versatile activators, such as UV light, ultrasound, microwaves, and alkalis. This offers the prospect of broadening the range of applications and enhancing controllability. In essence, our work represents the inception of a new chapter in rationally designing zeolite materials, enhancing versatility and substantial potential for future zeolite research and development.

## Supplementary Material



## Notes

Experimental details, methods and additional results of solid-state NMR spectra, XRD patterns, SEM images, TEM image with EDX mapping, N_2_ adsorption isotherms, and structure refinement data (PDF)
