# Supplementary material for: Targeted Dealumination via In Situ Activation of Persulfate in Size-Selective Zeolite Channels
Source: J Phys Chem Lett. 2025 Jun 5;16(23):5878–86. doi: 10.1021/acs.jpclett.5c00955 (PMC12169677; doi:10.1021/acs.jpclett.5c00955)
Supplement: Supplementary file 1 [file jz5c00955_si_001.pdf]

Supporting Information

Targeted dealumination via in-situ activation of persulfate in size-selective zeolite channels

*Youdong Xing,[a,b] Guangchao Li,[c,d] Yi Zhang,[c] Jochi Tseng,[e] Dong Fan,[f] Tianqi Cheng,[g] Yung-Kang Peng,[g] Tsz Woon Benedict Lo,[c,d] Keizo Nakagawa,[h] Shik Chi Edman Tsang,[*i] Molly Meng-Jung Li[*c,j]*

a. Interdisciplinary Institute of NMR and Molecular Sciences, Wuhan University of Science and Technology, Wuhan 430081, China.
b. Hubei Province for Coal Conversion and New Carbon Materials, School of Chemistry and Chemical Engineering, Wuhan University of Science and Technology, Wuhan 430081, China.
c. Department of Applied Physics, The Hong Kong Polytechnic University; Hong Kong 999077, China.
d. Department of Applied Biology and Chemical Technology, The Hong Kong Polytechnic University; Hong Kong 999077, China.
e. Diffraction and Scattering Division, Japan Synchrotron Radiation Research Institute, Spring-8, Sayo-gun, Hyogo 679-5198, Japan.
f. National Engineering Research Center of Lower-Carbon Catalysis Technology, Dalian National Laboratory for Clean Energy, Dalian Institute of Chemical Physics, Chinese Academy of Sciences, Dalian 116023, China.
g. Department of Chemistry, City University of Hong Kong, Hong Kong 999077, China.
h. Research Center for Membrane and Film Technology, Graduate School of Science, Technology, and Innovation, Kobe University, 1-1 Rokkodai, Nada, Kobe, 657-8501, Japan.
i. Wolfson Catalysis Centre, Department of Chemistry, University of Oxford; Oxford, OX1 3QR, UK.
j. Shenzhen Research Institute, The Hong Kong Polytechnic University, Shenzhen, Guangdong 518057, China.

*Correspondence
molly.li@polyu.edu.hk
edman.tsang@chem.ox.ac.uk

1. Experimental Sections and Characterizations

Samples preparation

A commercial sodium-form mordenite (Na-MOR) zeolite with SiO_2/Al_2O_3= 13 (CBV 10A, Zeolyst International) was used in this study. To remove any organic compounds, the sample was heated at 1°C per minute to 550°C under dry air and then held at this temperature for 5 hours. The resultant sample was further transformed into its ammonium form (NH_4-MOR) by ion exchange, which was carried out in 1.0 mol/L NH_4NO_3 solution with a liquid-to-solid ratio of 5. This conversion was performed three times at 80°C for 2 hours each time. Following each ion exchange process, the sample underwent thorough washing with deionized water. The resulting zeolite, NH_4-MOR, served as the initial sample for subsequent dealumination experiments with ammonium persulfate (APS) (Purchased from Aladdin with 98% purity).

Preparation of APS-introduced MOR

To prepare APS-introduced MOR, typically, 0.5 g of NH_4-MOR was dispersed in 3.5 wt % APS solution at room temperature (RT) for 9 hours under mechanical stirring. The resulting sample was then dried in a vacuum oven at RT for 5 hours to avoid the decomposition of APS during this stage. For characterization, the obtained APS-introduced sample was washed with deionized water several times, leading to the sample being identified as MOR-APS-RT.

Preparation of thermal-treated MOR samples with or without APS introduction

To dealuminate MOR with activated APS, the APS-introduced MOR was heated in a home-made flow reactor (see **Scheme S1**) at 80°C for 24 hours under a 200 mL/min N_2 flow with 17 Torr water vapor (saturated vapor pressure at RT). The products were thoroughly washed with deionized water several times and dried at 90°C overnight and were named MOR-APS-80C, respectively.

A control sample, thermal-treated MOR zeolite, was prepared by treating the pristine NH_4-MOR zeolite under the same conditions as MOR-APS-80C, excluding the contribution of heating on dealumination.

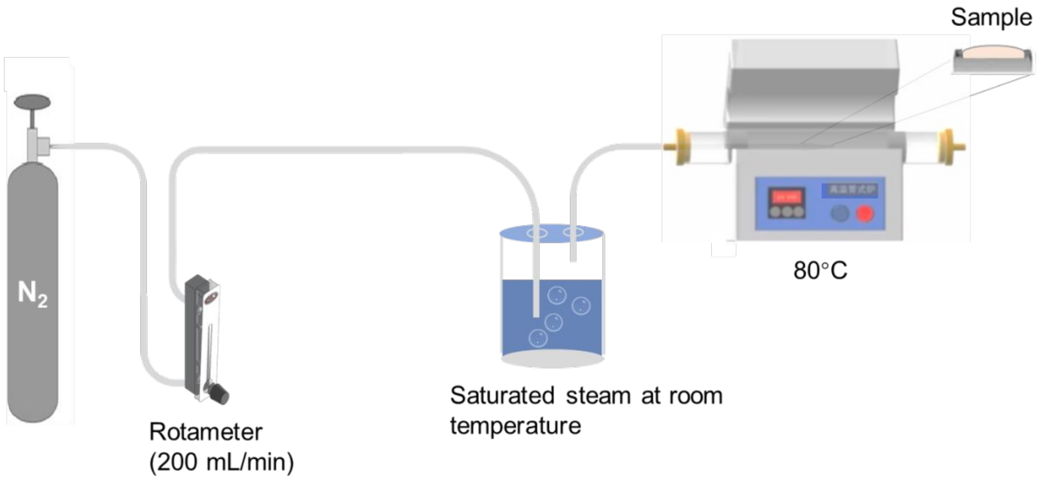

Scheme S1. The homemade flow reactor for the preparation of dealuminated samples.

2. Characterization Techniques

Sample treatments for solid-state nuclear magnetic resonance (SSNMR) experiments

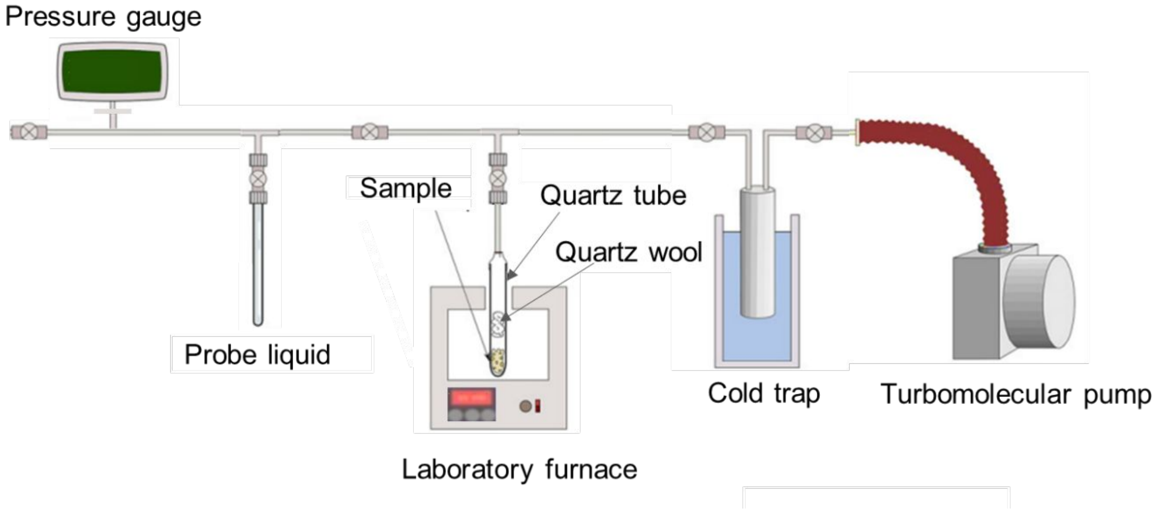

Scheme S2. The device of the vacuum system for sample dehydration and probe adsorption.

Dehydration of the sample was carried out in a vacuum line (**Scheme S2**). The heating temperature was ramped up at a rate of 1°C per minute to 450°C and then maintained at this temperature for 10 hours under a pressure below 10^{-3} Pa. The CD_3CN adsorption experiments were performed in the following steps. The dehydrated sample was exposed to oversaturated CD_3CN controlled by vapor pressure and sealed in the quartz tube at 60°C for 2 hours for saturated adsorption. Subsequently, the residual CD_3CN molecules were evacuated at RT for 0.5 hours to remove the weakly physisorbed molecules. Finally, the sample was transferred into the ZrO_2 rotor under a dry nitrogen atmosphere within a glovebox for NMR measurements.

Characterization

SSNMR
All SSNMR experiments were performed on a Jeol ECZ500R spectrometer equipped with a 3.2- and 8-mm magic angle spinning (MAS) probe, with resonance frequencies of 495.13, 129.02, and 98.37 MHz for ^{1}H, ^{27}Al, and ^{29}Si nuclei, respectively. All NMR lineshape simulations were carried out with the DMFIT software.

^{1}H MAS SSNMR spectra were acquired on a 3.2 mm probe with a spinning rate of 10 kHz, a pulse width of 2.43 μs (ca. π/2 pulse), a recycle delay of 5 s, and 32 scans. The chemical shifts of ^{1}H were referenced to solid adamantane at 1.7 ppm.

One dimensional (1D) ^{27}Al MAS SSNMR spectra were collected at a spinning rate of 12 kHz with a recycle delay of 0.5 s and 1024 scans. A small flip angle (π/12) associated with a short pulse duration (0.16 μs) was used to ensure the homogeneous excitation of the whole signals for the acquisition of a quantitative NMR spectrum.

2D ^{27}Al multiple-quantum (MQ) MAS z-filtering SSNMR experiments were conducted with the optimized pulse widths p1 = 5.0 μs, p2 = 1.4 μs, and p3 = 6 μs. The spectra were collected with 128 t1 increments and 256 scan accumulations for each t1 increment. The SSNMR parameters

determined from the ^{27}Al MQ MAS SSNMR spectra were used to simulate the ^{27}Al MAS SSNMR spectra for quantitative analysis on the different Al sites. The chemical shifts of ^{27}Al were referenced to a 1.0 M aqueous $Al(NO_3)_3$ at 0 ppm.

^{29}Si MAS NMR spectra were acquired at a spinning rate of 4 kHz on an 8 mm probe head by a single π/2 pulse with 7.61 μs width and a recycle delay of 60 s and 24 scans.

^{29}Si(^{1}H) cross-polarization (CP) MAS spectra were performed with a radio frequency (rf) field of 23 and 18 kHz for ^{29}Si and ^{1}H, respectively, with a contact time of 4 ms. ^{1}H TPPM decoupling was applied during the ^{29}Si signal acquisition (pulse length 8.67 μs, rf field ca. 33 kHz). ^{29}Si chemical shifts were referenced to kaolinite.

Synchrotron powder X-ray diffraction (SXRD)
High-resolution SXRD data were collected at beamline BL02B2 at SPring-8, Japan. The energy of the incident X-ray flux was set at 17 keV. The wavelength (0.7002946(34) Å) and the 2θ-zero (0.00222(4)°) point were calibrated using a diffraction pattern obtained from a high-quality silicon powder (NIST SRM640c). The pristine MOR and APS-treated zeolites were loaded in a 0.5 mm borosilicate glass capillary and then sealed using a flame. SXRD data of the samples were collected using the MYTHEN detectors at -173°C to minimize the thermal motion of APS molecules probably disordering APS locations in the zeolite channels. Each SXRD pattern was collected in the 2θ range 2-80° with 0.006° data binning for 450 s for each MYTHEN-2θ-step. This offers a high signal-to-noise ratio for better data resolution.

Temperature-dependent SXRD experiment was conducted over a temperature range of 0°C to 125°C at a ramping rate of 10°C per minute. SXRD patterns of the APS-introduced MOR sample were recorded at every 25 °C temperature step in the 2 θ range 2-80° with 0.006° data binning. Each SXRD pattern was collected for 450 s.

The lattice parameters were determined using Le Bail and Rietveld refinement analyses of the diffraction patterns with TOPAS software. The starting coordinates for the refinement were based on the MOR zeolite model in the official Database of the International Zeolite Association (IZA). Background curves were fitted using a Chebyshev polynomial with an average of 15 coefficients, and the TCHZ peak type was employed to describe the diffraction peaks. Scale factors and lattice parameters were allowed to vary for all the histograms. The final refined structural parameters for each data histogram were obtained using the Rietveld method, incorporating fractional coordinates (x, y, z) and isotropic displacement factors (B_{eq}) for all atoms. In addition, the goodness-of-fit values (R_{wp} and GOF = R_{wp}/R_{exp}) where R_{exp} denotes the quality of the data, were utilized to evaluate the quality of fit, with a R_{wp} value close to R_{exp} indicating a good fit.

(i) Refinement of framework atoms
To prevent a miscalculation of the structure that could result from changes to the entire framework upon introduction of APS into the zeolite channels, the positions of the framework atoms were refined separately before performing the refinement of the entire structure with the guest molecules. Since the guest molecules used in the study do not contain heavy atoms, it is reasonable to assume that they have minimal influence on the low 2θ angle reflections. Therefore, the fractional coordinates and B_{eq} values of the framework atoms (Si and O) were fixed, and all other parameters were refined over the 2θ range of 2-45°.

(ii) Fourier analysis

Following the refinement of framework atoms, Fourier analysis was employed to identify the positions in the framework with the highest remaining electron density over the 2θ refinement range.

(iii) Inclusion of guest molecules
Based on the results of the Fourier analysis, the positions of the guest persulfate molecules in the MOR were determined by treating them as rigid bodies. The Z-matrices of the guest molecule rigid bodies were refined, while keeping the fractional coordinates of the framework atoms fixed. The APS-introduced MOR samples were refined using two rigid body Z-matrices that describe the APS at fixed bond distances and angles. The refinement process included a simulated annealing step using the Rietveld method, with the fractional coordinates of the framework atoms held constant. The simulated annealing technique ensured accurate determination of the APS sites.

After identifying the number and positions of the residence sites, the site occupancy factors (SOFs) were refined. Subsequently, the relevant parameters were subjected to repeated simulated annealing refinement for an hour to ensure convergence to the global minimum. The lowest R_{wp} and GOF values were indicative of the global minimum.

In cases where the derived crystal structures were chemically questionable, such as when the guest molecules were located in close proximity to the framework, a thorough examination of the parameters was conducted before repeating the refinement procedure. Multiple criteria were employed to ensure the high quality and reliability of the refinement, including (a) attainment of the global minimum, (b) chemical suitability of the derived crystal structure, (c) reasonably low systematic error values for all refined parameters, and (d) sensible SOF and B_{eq} values. The B_{eq} values were constrained as follows: all T-sites (T = Al, Si) shared the same value, and the values for the O-sites were twice the B_{eq} value for the T-sites.

The atomic and crystallographic parameters are summarized in **Tables S1-S4**. The position errors of APS were estimated from the percentage errors of the translation and rotation axes of the rigid bodies.

All the structure models refined are drawn using the software Materials Studio.

High-energy X-ray total scattering (HEXTS) measurements
In-situ HEXTS measurements were carried out at the BL08W beamline of SPring-8 in Japan using high-energy X-rays (114.03 keV, λ = 0.1087 Å). The experimental set-up of the in situ HEXTS measurements was shown in **Scheme S3**. The 2 cm of sample powders (APS-introduced MOR zeolite) were packed into a Kapton capillary between two plugs of glass wool, which was fixed on a homemade sample holder. The testing conditions were consistent with the experimental conditions used to prepare the thermal-treated MOR sample (see Experimental sections). Nitrogen gas generated using a nitrogen gas generator was passed through a water bubble to carry water vapor at RT, which was then passed over the heated samples in an 80°C heater. Data is collected with a 2-dimensional area detector (Perkin-Elmer 1621En) at time intervals of 2 hours for the first 4 hours, followed by 4-hour intervals and were recorded for 12 hours in total. The collected image data were integrated to $I(Q)$ based on the instrumental calibration using NIST SRM 674b CeO_2 and masking. The maximum Q ($Q = 4\pi\sin\theta/\lambda$), Q_{max}, collected in this study was 18 Å. According to the adsorption corrections of materials and compton profile, the $S(Q)$ were calculated and subsequently transformed to the $G(r)$ data *via* Fourier transformation. All the data processing was performed using the software DAWN.[1]

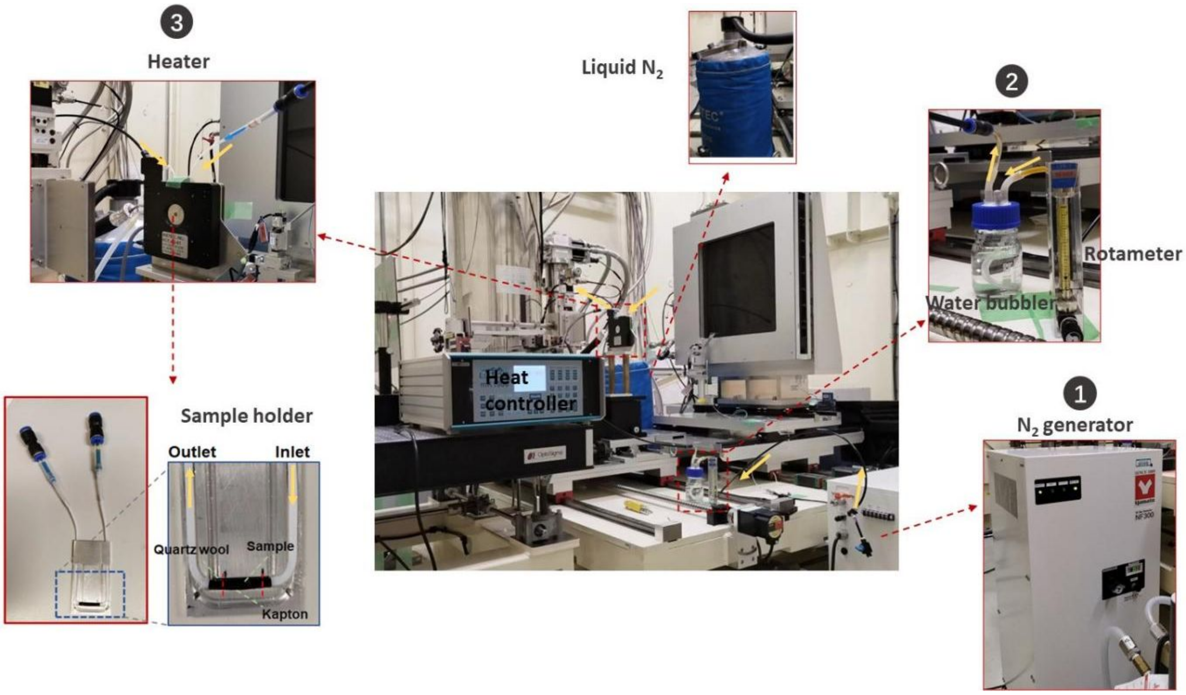

Scheme S3. The experimental set-up of the *in situ* HEXTS measurements.

UV-vis absorption
All the UV-vis absorption spectra were recorded on a UV-Vis spectrophotometer (Shimadzu UV-2550) equipped with quartz cuvettes of 1 cm light path.

Electron paramagnetic resonance (EPR)
All EPR measurements were conducted at RT on an ADANI SpinscanX spectrometer, operating at 100 kHz field modulation using 150 microtesla (μT) as the modulation amplitude. A mass of 4 mg of APS-introduced MOR, was carefully placed inside a glass capillary having a 1 mm inner diameter. Subsequently, the capillary was transferred to a water bath maintained at either RT or 80°C for 4 hours. Following this, a 50 μL volume of 20 wt% 5,5-dimethyl-1-pyrroline-N-oxide solution was injected into the glass capillary to capture the radicals generated from the activated APS for the EPR measurements.

Field Emission Scanning Electron Microscope (FESEM)
The microstructure and morphology of the pristine MOR and APS-treated samples were measured by Field Emission Scanning Electron Microscope (FESEM, Tescan MAIA3).

Transmission electron microscopy (TEM) and energy-disperse X-ray (EDX) mapping
TEM and EDX mapping were carried out on a JEOL JEM-F200 at 200 kV, using the copper grid. The zeolite was dispersed in ethanol with 5 minutes of ultrasonic before analysis.

Laboratory X-ray diffraction (XRD)
All XRD diffraction patterns were collected on a Rigaku SmartLab 9 kW diffractometer using Cu Kα radiation (λ = 1.5418 Å) in the 2θ range 5-60° with scanning steps of 0.08° per second.

X-ray photoelectron spectroscopy (XPS)
X-ray photoelectron spectroscopy measurements were conducted using a Thermo Fisher Esca Lab.

The spectra were calibrated by observing the adventitious C 1s peak at around 284.8 eV for all the tested samples.

Nitrogen adsorption/desorption isotherm

Nitrogen adsorption and desorption measurements were conducted using a Micromeritics ASAP 2020 automated gas adsorption analyzer at -196°C. Before analysis, the samples were outgassed at 350°C for 10 hours. The specific surface area and pore volumes were determined by Brunauer-Emmett-Teller and t-plot methods, respectively.

X-ray fluorescence spectroscopy (XRF)

The chemical composition of samples was determined using a Rigaku ZSX Primus III+ XRF spectrometer.

Catalytic evaluation

The carbonylation of dimethyl ether (DME) to methyl acetate (MA) was carried out in a stainless-steel tubular fixed-bed reactor under the following conditions: 2 MPa, 473 K, a gas hourly space velocity (GHSV) of 3600 mL/g/h, and a reactant gas mixture of DME/CO/N_2 (5/35/60). Typically, 0.2 g of catalyst was packed into the reactor and pretreated with nitrogen at 773 K for 1 hour. After cooling the catalyst to 473 K, the system was gradually pressurized to 2 MPa with the DME/CO/N_2 mixture. The outlet gas was analyzed online using a gas chromatograph (Agilent 7890B) equipped with a flame ionization detector (FID) and a PLOT-Q capillary column.

The DME conversion and MA selectivity were calculated based on carbon atoms using the following equation:

$$\text{DME conversion} = \frac{2C_{MA}+C_{methanol}+\sum_i iCi}{2C_{DME}+2C_{MA}+C_{methanol}+\sum_i iCi} \times 100\%$$

$$\text{MA selectivity} = \frac{2C_{MA}}{2C_{MA}+C_{methanol}+\sum_i iCi} \times 100\%$$

Where C_{MA}, C_{DME}, and $C_{methanol}$ represent the moles of MA, DME, and methanol at the outlet, respectively. C_i represents the moles of individual hydrocarbon products at the outlet.

3. Supplementary Figures and Tables

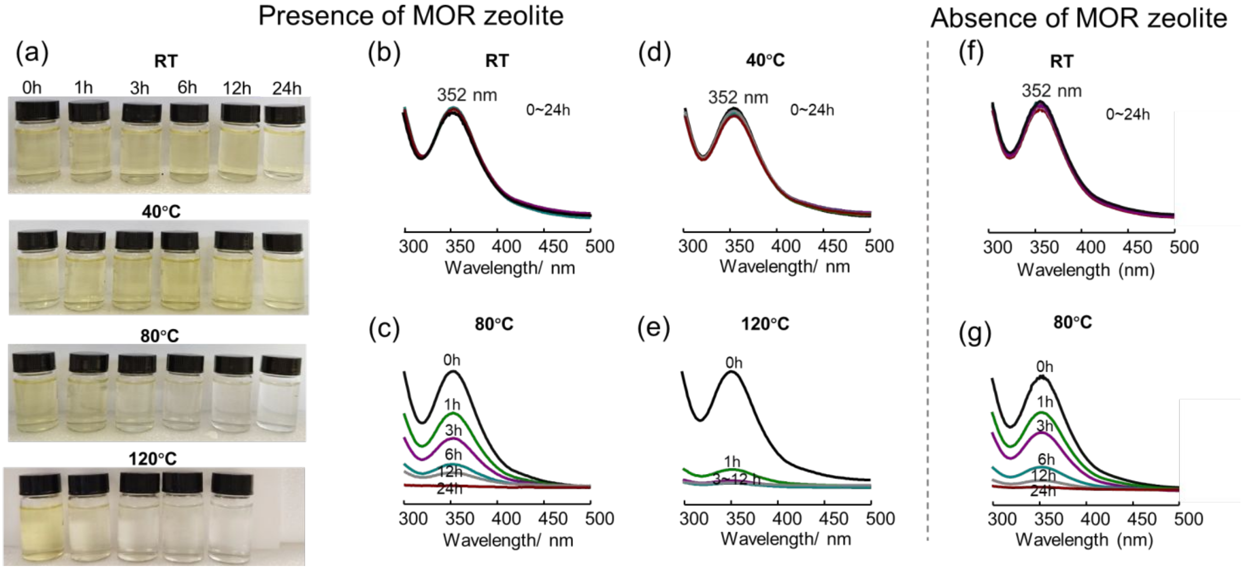

Figure S1. (a) The coloration of the mixture APS/MOR over time under RT, 40°C, 80°C and 120°C. The UV-vis absorption spectra of the mixture set **(b-e)** and APS solution with the absence of MOR zeolite **(f-g)**.

To determine the optimal temperature to introduce and activate APS, we employed the UV-vis spectrophotometric method to monitor the changes in persulfate concentrations/decomposition over time at various temperatures. This was accomplished by measuring the formation of a yellow iodine color resulting from the reaction between persulfate and KI ($S_2O_8^{2-} + 2I^- \rightarrow 2SO_4^{2-} + I_2$). The detection method refers to Liang's work.[2]

4 g of KI was added to a 40 mL $NaHCO_3$ (0.5 wt%) solution to prepare the color indicator. Note that the inclusion of $NaHCO_3$ serves to remove oxygen from the solution to prevent the air oxidation of iodide.[2] Four identical suspensions of 0.5 g MOR and 3.5 wt% APS in deionized water were heated at RT, 40°C, 80°C and 120°C respectively. At specific heating times, 2 μL of each suspension was extracted and added to the prepared KI indicator solution.

Figure S1a illustrates the coloration of the mixture APS/MOR after the addition of the KI indicator over time under RT, 40°C, 80°C and 120°C. At RT and 40°C, the mixture's color exhibits invisible changes over time while upon exposure to higher temperatures (80°C and 120°C), the yellow hue transforms into a colorless solution over time. This observation indicates that APS concentration changes over time under different temperatures. **Figures S1b-e** depict the UV-vis absorption spectra of the mixture set, revealing a characteristic absorption peak at 352 nm.[2] The peak intensity exhibits no substantial variation over 24 hours at RT, suggesting the concentration of APS remains constant and the stability of APS at this temperature. In contrast, the peak intensity decreases with enhanced temperatures (40°C, 80°C and 120°C) at different rates, indicating the variation of APS concentration and the temperature-controlled decomposition process of APS. The change in relative APS concentration (C/C_0) over time at each temperature, depicted in **Figure 2a,** was obtained from the peak intensity of the mixture relative to that at the start time. In this work, we selected 80°C for attaining mild and controllable activation/decomposition of APS, and RT (remaining APS inertness) for the non-destructive introduction of APS into the zeolite.

Note that we also conducted supplementary control experiments without the addition of zeolite to investigate the influence of zeolite on the activation of APS. In these experiments, we examined the APS solution alone and observed a similar change pattern to that of the APS/MOR zeolite mixture. Specifically, we observed a consistent intensity of the 352 nm peak over time at RT, while a decrease was observed at 80°C (**Figure S1f** and **1g**). Therefore, based on the control experiment, we can conclude that high temperature is the primary contributor to the activation/decomposition of APS, regardless of the presence of zeolite.

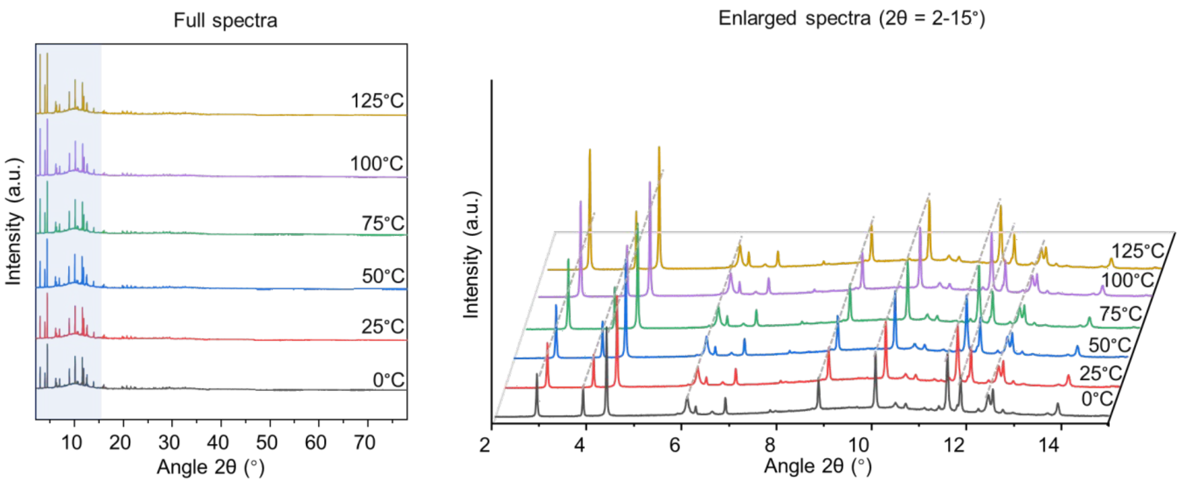

Figure S2. Temperature-dependent SXRD data of the APS-introduced MOR zeolite from 0°C to 125°C.

To investigate the thermally triggered activation process of APS, a temperature-dependent high-resolution SXRD experiment was conducted. In **Figure S2**, the SXRD profiles (2θ < 6°) of the APS-introduced MOR zeolite show minimal changes within the low-temperature range (0°C to 25°C). However, as the temperature increases, there is a noticeable and consistent increase in the intensity of the peaks corresponding to the MOR structure. This change in peak intensity reflects the alteration in zeolite pore filling as APS decomposed in situ at higher temperatures.[3-4] Note that the intensity of peaks in high 2θ angle (> 6°) remains almost constant, indicating a short-range order in the MOR zeolite structure during the APS activation.

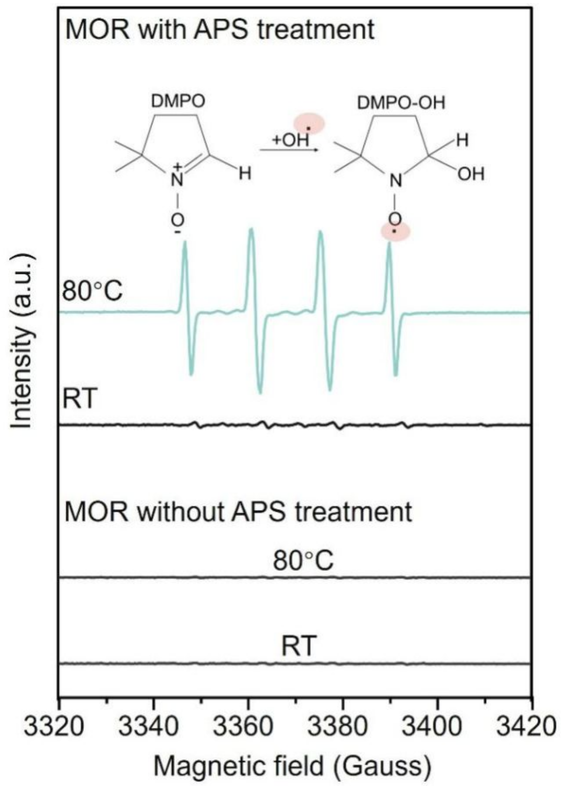

Figure S3. Comparative EPR analysis of radical generation in APS-treated vs. untreated MOR zeolite at RT and elevated (80°C) temperatures.

To verify the role of APS in generating radicals, we performed blank experiments on MOR zeolites at both RT and 80°C, without the inclusion of APS. These blank experiments showed no radical signals regardless of temperature (RT or 80°C). This marked contrast with the APS-treated counterparts—which exhibited distinct radical signatures exclusively at 80°C—definitively establishes the temperature-dependent activation mechanism of persulfate as the critical driver for selective framework dealumination.

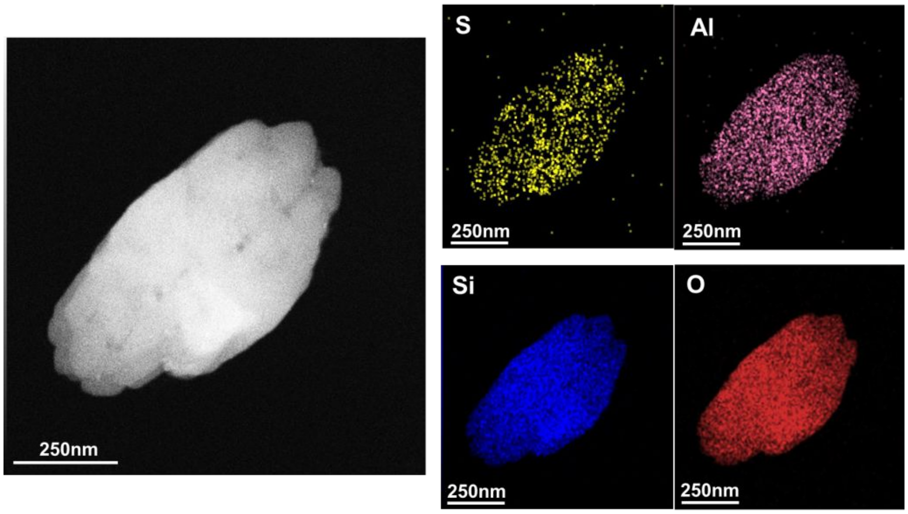

Figure S4. TEM image and EDX elemental mapping (S, Al, Si, and O) in the APS-introduced MOR zeolite, which shows a uniform distribution of S element within the crystals, confirming the even penetration of persulfate molecules into the zeolite.[5]

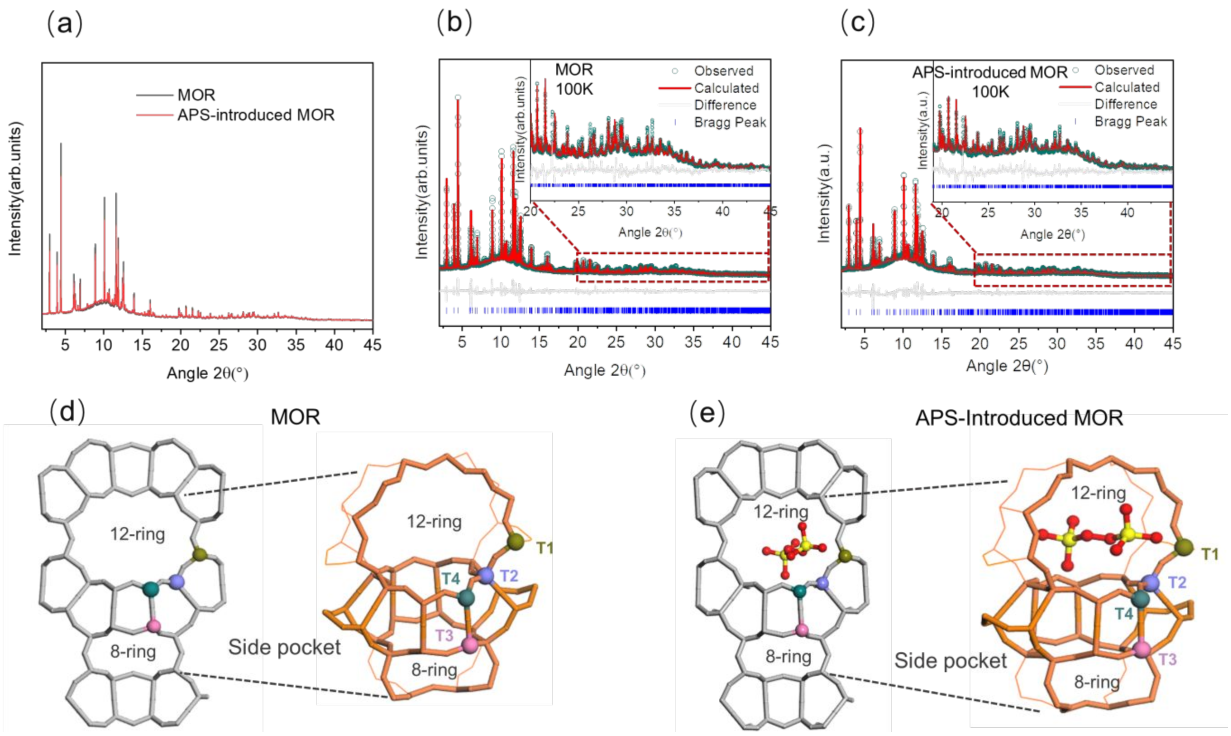

Figure S5. (a) The overlapped SXRD data of the pristine MOR and APS-introduced MOR zeolites and **(b-c)** SXRD patterns and Rietveld refinement profiles of both samples. SXRD data in the range of 19-45° are expanded to illustrate the quality of the Rietveld refinement. **(d-e)** Crystallographic models of the samples based on SXRD Rietveld refinement.

The SXRD patterns **(Figure S5a)** show a significant difference in Bragg's peak intensities between the pristine MOR and APS-introduced MOR zeolites, which is attributed to the introduction of guest persulfate into the zeolite channels. This is supported by the analysis of the good refined diffraction data of the samples (**Figures S5b-5c)**, with the small discrepancies in the grey difference profiles and low-reliability factors (R_{wp}, R_{exp} and λ^2). The refined structure model shows that the introduced APS molecule exclusively resides in the 12-ring channel and is oriented parallel to the channel, facing the 12-ring window (**Figure S5e**). The detailed structural parameters are summarized in **Tables S1-S4**.

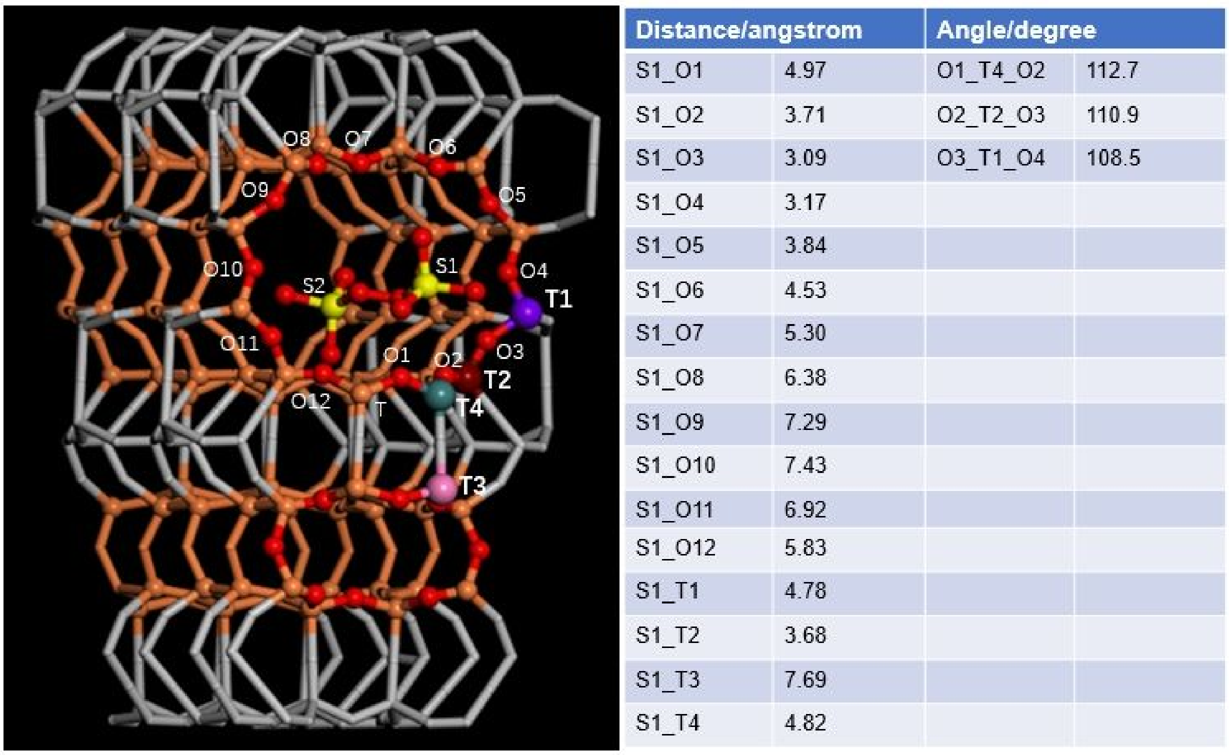

Distance/angstrom		Angle/degree	
S1_O1	4.97	O1_T4_O2	112.7
S1_O2	3.71	O2_T2_O3	110.9
S1_O3	3.09	O3_T1_O4	108.5
S1_O4	3.17		
S1_O5	3.84		
S1_O6	4.53		
S1_O7	5.30		
S1_O8	6.38		
S1_O9	7.29		
S1_O10	7.43		
S1_O11	6.92		
S1_O12	5.83		
S1_T1	4.78		
S1_T2	3.68		
S1_T3	7.69		
S1_T4	4.82		

Figure S6. Crystallographic model based on SXRD Rietveld refinement. Symmetry in adsorption sites is disregarded for clarity**.** The selected interatomic distance between the S of the persulfate and the atoms in different oxygen/T sites in the 12-ring channel, and the angles related to T sites in the 12-ring channel.

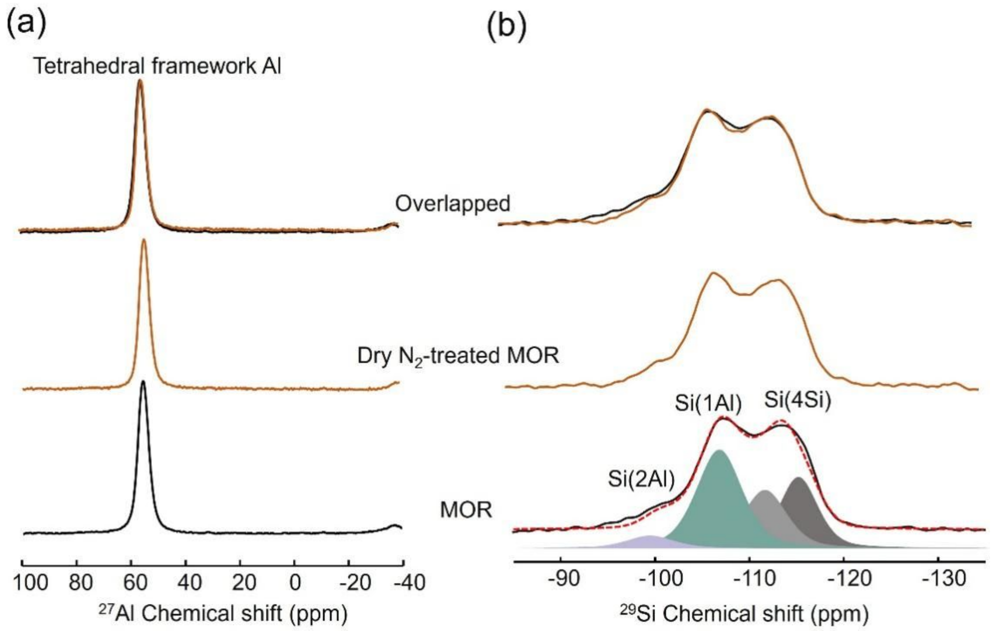

Figure S7. Weight-normalized **(a)** ^{27}Al and **(b)** ^{29}Si MAS SSNMR spectra of the pristine MOR and dry N_2-treated MOR zeolite at 80°C.

To rigorously validate the necessity of moisture, we performed parallel control experiments under dry N_2 flow. As shown in **Figure S7**, these dry treatments resulted in negligible Al removal as evidenced by the identical pattern between the treated MOR and the pristine MOR in both ^{27}Al and ^{29}Si MAS NMR spectra, unambiguously demonstrating that water is indispensable for framework etching.

(a)

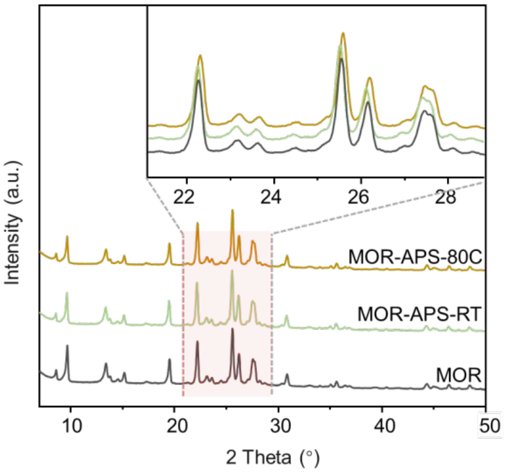

(b)

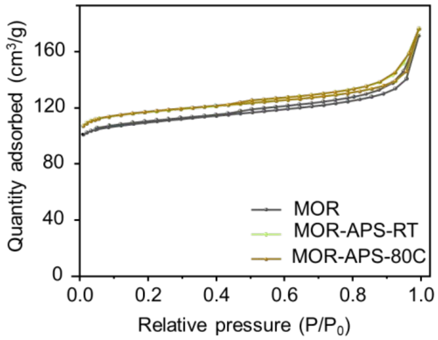

(c)

Sample	[a]S_{BET} (m^2 g^{-1})	[b]S_{meso} (m^2 g^{-1})	[c]V_{mic} (cm^3 g^{-1})	[d]V_{meso} (cm^3 g^{-1})	[e]V_{total} (cm^3 g^{-1})
MOR	357	59	0.15	0.12	0.27
MOR-APS-RT	344	69	0.14	0.14	0.28
MOR-APS-80C	358	61	0.15	0.12	0.27

[a] BET specific surface area. [b] Mesopore surface area, t-plot. [c] Micropore volume, t-plot. [d] Mesopore volume, (V_{total}-V_{mic}). [e] Total volume.

Figure S8. (a) XRD patterns, **(b)** N_2 physisorption isotherms of the pristine MOR and APS-treated MOR zeolites and **(c)** their textural properties. XRD data in the range of 21-29° are expanded to illustrate the nearly unchanged peak intensity before and after the APS treatment.

All APS-treated samples maintain a distinct MOR structure with high crystallinity similar to that of the pristine MOR, as evidenced by the characteristic peaks with small changes in the peaks' intensity (**Figure S8a)**. Besides, APS-treated MOR samples display comparable isotherms (a combination of types I and IV isotherms) to pristine MOR in **Figure S8b**, with a steep increase in N_2 adsorption in the low-pressure region (P/P_0 < 0.1) and small hysteresis loop. A detailed description of the textural properties of these samples is shown in **Figure S8c**, where both specific surface area and pore volume show a small difference between the pristine MOR and APS-treated MOR. This suggests their similar porous structures. Therefore, it can be concluded that APS treatment on the MOR zeolite does not induce obvious structure destruction to its macroscopic crystalline and porous structures.

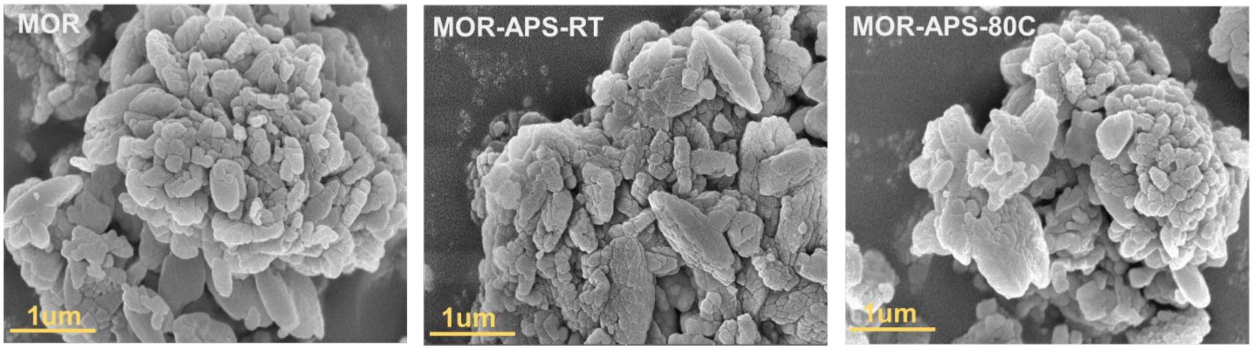

Figure S9. SEM images of the pristine MOR and APS-treated MOR zeolites show no obvious changes in the surface and morphology of the crystals before and after APS treatment.

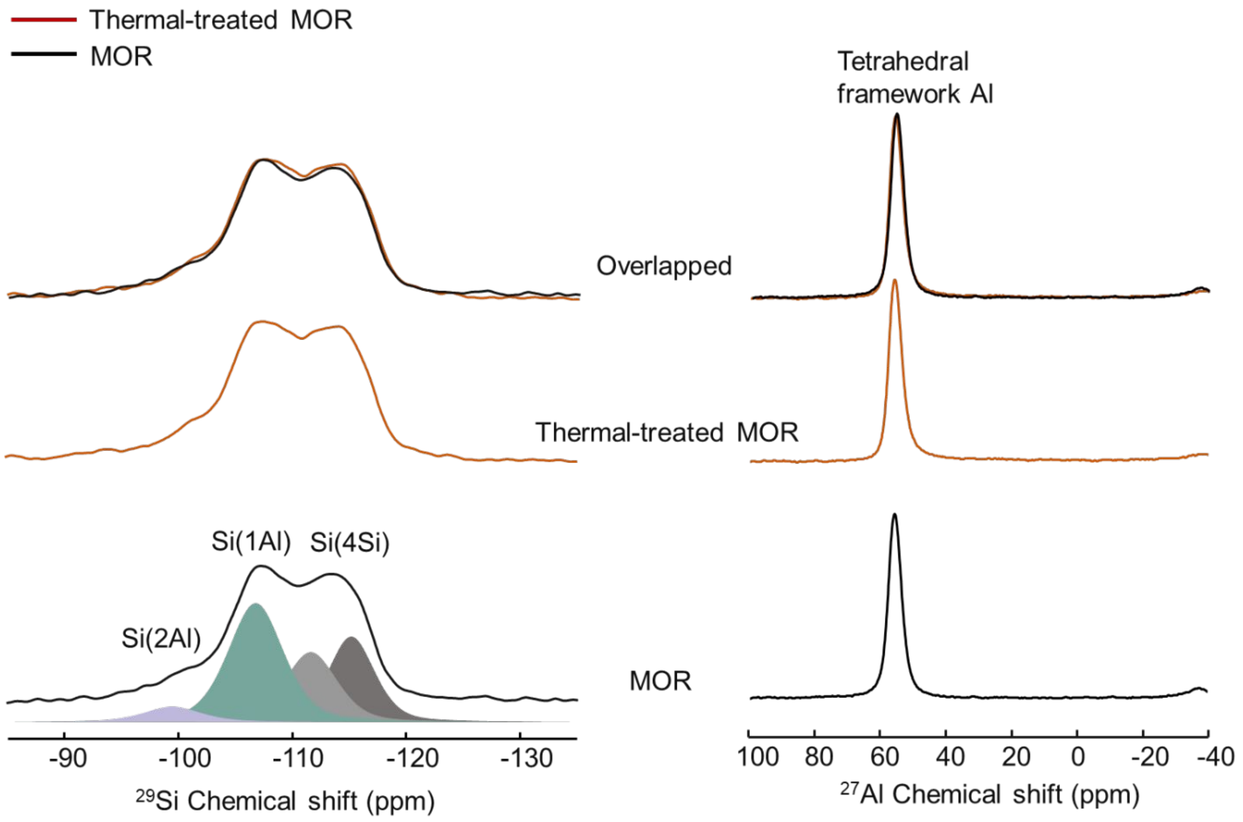

Figure S10. Weight-normalized **(a)** ^{29}Si and **(b)** ^{27}Al MAS SSNMR spectra of the pristine MOR and thermal-treated MOR zeolite at 80°C. The Si signals were deconvoluted using the DMFIT software.

To exclude the contribution of heating to the removal of framework Al in our proposed dealumination method, ^{29}Si and ^{27}Al MAS SSNMR experiments were employed to assess the concentration of framework atoms in the pristine MOR and thermal-treated MOR zeolite at 80°C for 24 hours (refer to experimental sections). **Figure S10a** shows the ^{29}Si MAS SSNMR spectra of both samples, in which distinctive signals at 110~116 ppm, -106 ppm and -98 ppm correspond to Si(4Si), Si(1Al) and Si(2Al) species, respectively.[6] Thermal-treated MOR exhibits an identical pattern to that of the pristine MOR, indicating that there are no significant changes in these framework species after the thermal treatment and dealumination does not occur in the single thermal treatment process. This is further supported by ^{27}Al MAS SSNMR spectra (**Figure S10b**) where a single framework Al signal at 55 ppm is identified in both samples with the same signal intensity.[7] Consequently, heating as a potential contributor to the removal of framework Al can be excluded.

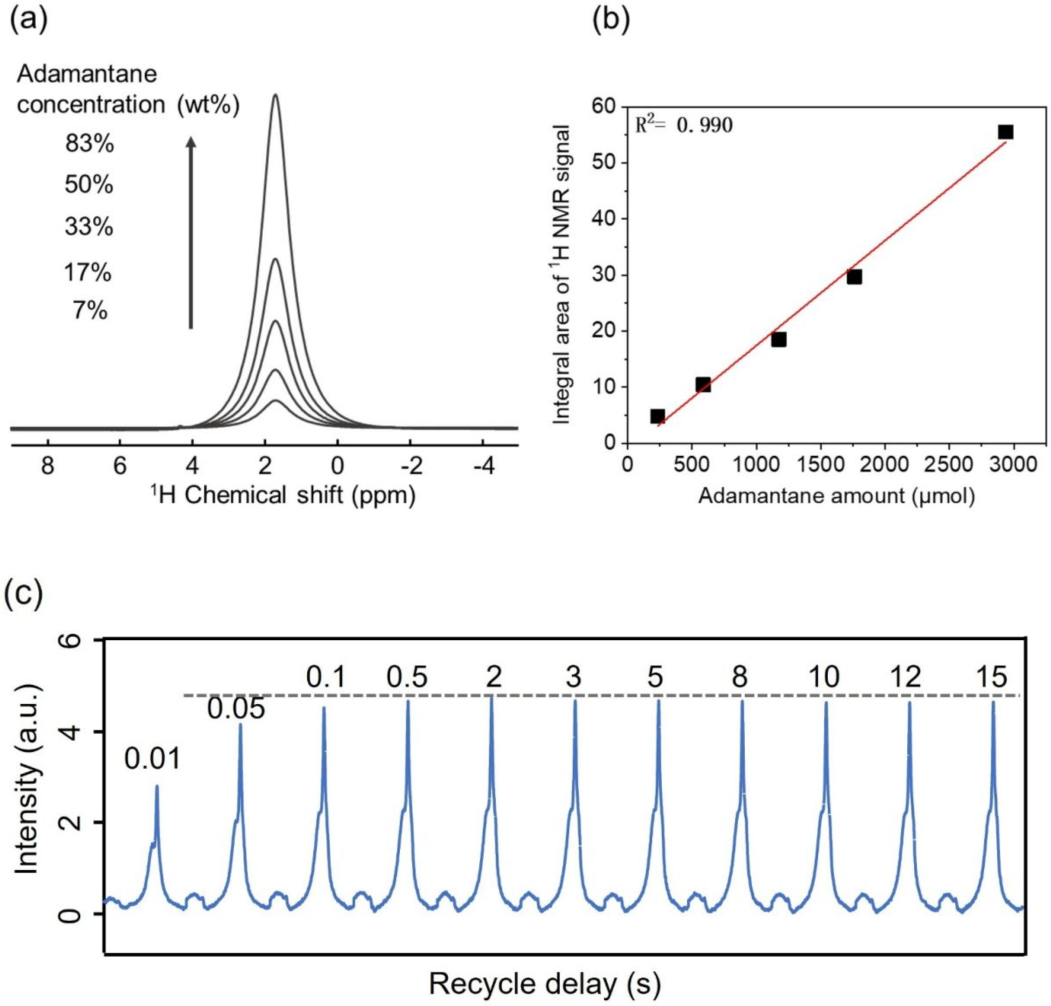

Figure S11. (a) 1D ^{1}H MAS NMR spectra of standard samples mixing with a known amount of adamantane and NaCl (a total of 30 mg). **(b)** ^{1}H NMR calibration line established by running the set of standard samples for the quantitative analysis of the concentration of Brønsted acid sites in the investigated samples via the peak area in the weight-normalized ^{1}H NMR spectra (**Figure 4d**). **(c)** ^{1}H MAS NMR spectra of dehydrated pristine MOR acquired with variable recycle delays (0.01–15 s). The intensity of Brønsted acid protons (δ~4.0 ppm) stabilized after 2 s, confirming that the selected 5 s delay exceeds the normally used 5×T_1 for complete magnetization recovery, thereby ensuring quantitative reliability.

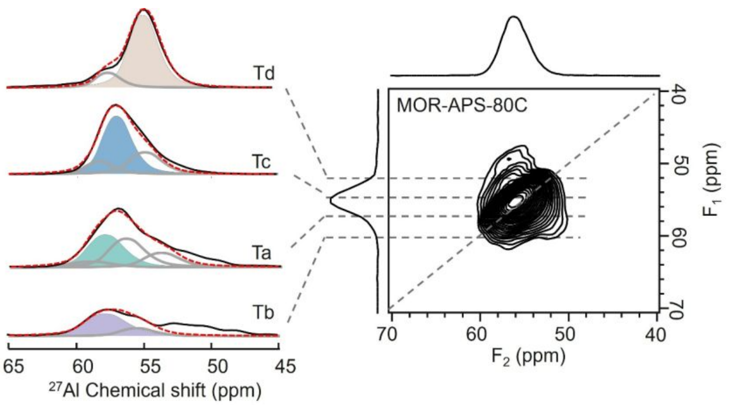

Figure S12. ^{27}Al MQ MAS SSNMR spectrum of the APS- MOR at 11.6 T and the deconvoluted slices extracted from the F1 dimension.

2D ^{27}Al MQ MAS SSNMR experiments were conducted to alleviate the quadrupolar effect of ^{27}Al and distinguish different Al T-sites in the MOR and APS-dealuminated zeolites. In an MQ MAS experiment, anisotropic quadrupolar interactions are refocused and an isotropic dimension is present in the F1 axis of the spectrum. The second-order quadrupolar line shape is only reflected in the F2 dimension. **Figure S12** shows the MQ spectrum of the MOR-APS-80C zeolite and the extracted representative slices in the isotropic F1 dimension, which demonstrates distinguished Al environment by various chemical shifts. By simulating these slices, the isotropic chemical shifts (δ_{iso}) and quadrupole interaction constants (C_Q) were extracted (**Table S5**), which were used to fit 1D ^{27}Al MAS SSNMR spectra for quantitative analysis, see **Figure 4c**.

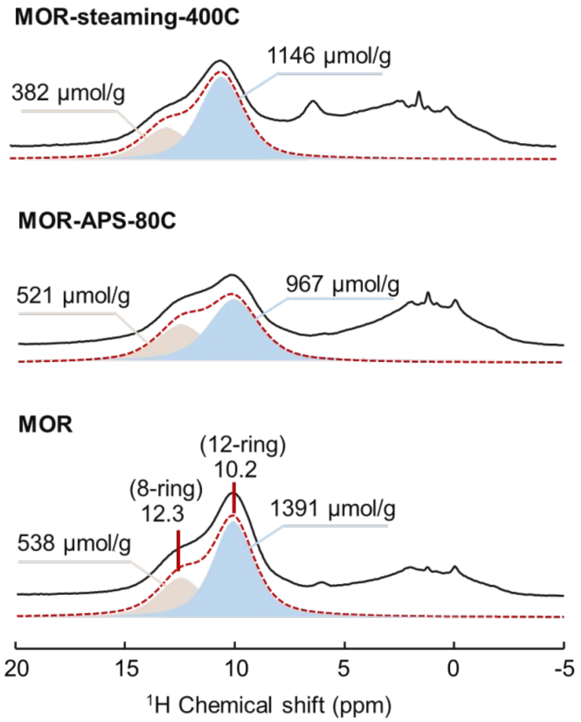

Figure S13. The deconvoluted ^1H MAS SSNMR spectra of the pristine MOR and APS-treated MOR and steamed MOR at 400℃ adsorbed CD_3CN.

The high-temperature steamed MOR at 400℃ was prepared to compare the differences in dealumination between the conventional high-temperature steaming method and our proposed persulfate-based method. Quantitative analysis of the CD_3CN probe-assisted ^1H SSNMR spectra (**Figure S13**) reveals a comparable relative amount of dealumination in both the conventional high-temperature steamed-MOR (21%) and the persulfate-treated MOR (23%) when compared to the pristine MOR. The dealumination induced by the conventional high-temperature steaming occurs non-selectively, as evidenced by the significant reduction of Al atoms located in both the 8-ring and 12-ring channels. Specifically, for the MOR-steaming-400C sample, the quantity of framework Al atoms within the 12-ring channels decreases by 245 μmol/g and the 8-ring Al decreases by 156 μmol/g compared to the pristine MOR sample. This non-selective dealumination observed with the high-temperature steaming method aligns with findings reported in previous literature on MOR high-temperature dealumination study.[8] In contrast, the persulfate-induced dealumination occurs exclusively in the 12-ring channels, resulting in a reduction of Al content from 1391 μmol/g to 967 μmol/g, whereas the Al content in the 8-ring channels remains constant.

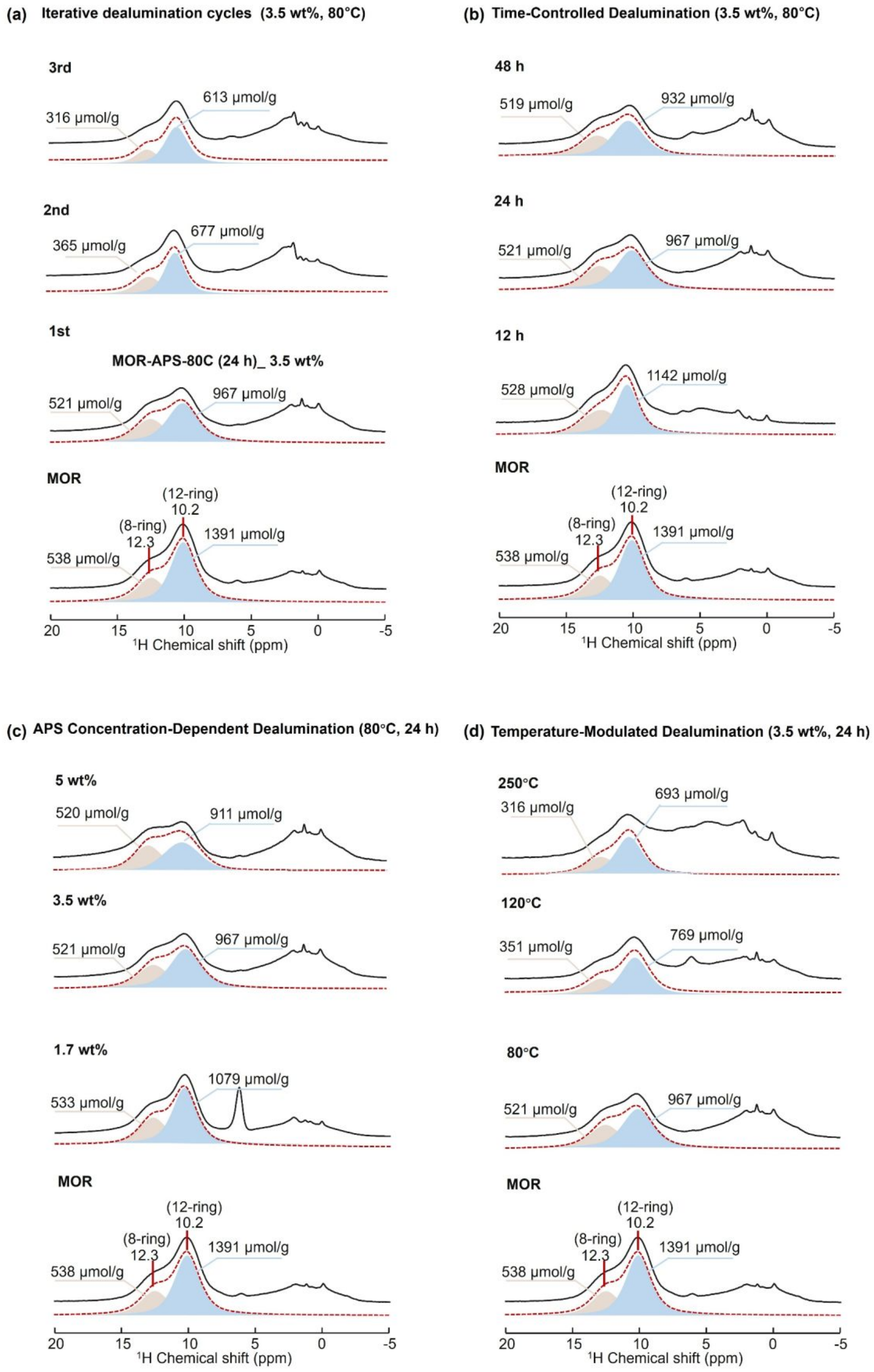

Figure S14. Deconvoluted ^{1}H MAS SSNMR spectra with CD_3CN probing for quantitative analysis of 12- and 8-ring Al removal in MOR zeolite via APS-mediated dealumination under (a) iterative dealumination cycles, (b) activation time, (c) controlled APS concentration and (d) activation temperature.

To systematically evaluate the method's tunability, we conducted parametric studies, including iterative dealumination cycles, controlled APS concentration and activation time/temperature conditions.

Cycling treatments (Figure S14a): The initial treatment resulted in 22% Al removal exclusively from the 12-ring channels, with complete preservation of 8-ring Al. Subsequent cycles demonstrated cumulative effects: Al extraction in 12-ring channels progressed to 37% (second cycle) and 40% (third cycle), accompanied by minor 8-ring Al loss (9% after the second cycle and 12% after the third). Importantly, the selectivity for 12-ring Al remained pronounced throughout, with its removal rate consistently exceeding that of 8-ring Al by more than threefold. This suggests that iterative cycling allows for tunable 12-ring Al extraction. However, at higher Al extraction amount (50% removal), some 8-ring Al loss occurred, and the effectiveness of dealumination decreased significantly after 2nd cycle. This indicates the need for further parameter optimization to improve selectivity at high extraction levels. Nonetheless, these results underscore the method's effectiveness for selective framework modification.

Time-dependent control (Figure S14b): Extending the APS activation time from 12 to 24 h at a fixed APS concentration (3.5 wt%) reduces the 12-ring Al content. Specifically, the 12-ring Al content decreases from 1391 μmol/g in the parent MOR to 1142 μmol/g (accounted for 13% Al removal) after 12 h and further to 967 μmol/g (22% removal) after 24 h. In contrast, the 8-ring Al content remains nearly stable, with minimal changes from 538 to 521 μmol/g (0.9% removal). Beyond 24 h, the etching efficiency declines sharply, yielding only an additional 37 μmol/g Al removal after 48 h (2% of total Al content). This trend aligns with UV-vis data, indicating APS exhaustion at 24 h, which terminates the reactive species generation and suppresses further dealumination (**Figure 2a** in the original manuscript).

Concentration modulation (Figure S14c): Modulating APS concentration from 1.7 to 5 wt% selectively reduces 12-ring Al from 1391 to 911 μmol/g (25% Al removal), while 8-ring Al shows negligible removal (0.9%) across all concentrations.

Thermal-dependent control (**Figure S14d**): At 80°C, 22% of Al was removed exclusively from the 12-ring channels, while 8-ring Al remains preserved. Elevating the temperature to 120°C and 250°C progressively enhances Al removal in the 12-ring channels to 32% and 36%, respectively, while Al removal in the 8-ring channels remains markedly lower at 10% and 12%, maintaining a consistent threefold higher efficiency for 12-ring Al extraction. However, partial removal of 8-ring Al under more aggressive dealumination conditions appears unavoidable, as similarly observed in $SiCl_4$-based methods where substantial 8-ring Al loss occurs at high dealumination levels[6]. This underscores the necessity of precise control over dealumination parameters to optimize site-selective framework modification.

These results demonstrate that our method exhibits high parameter sensitivity, enabling precise control over an effective extent of dealumination (up to 40% Al removal in 12-ring channels in the tested treatment parameters, while suppressing Al lose in the 8-ring channels). Moreover, it shows considerable potential for further optimization of both efficiency and site selectivity through the refined modulation of treatment parameters.

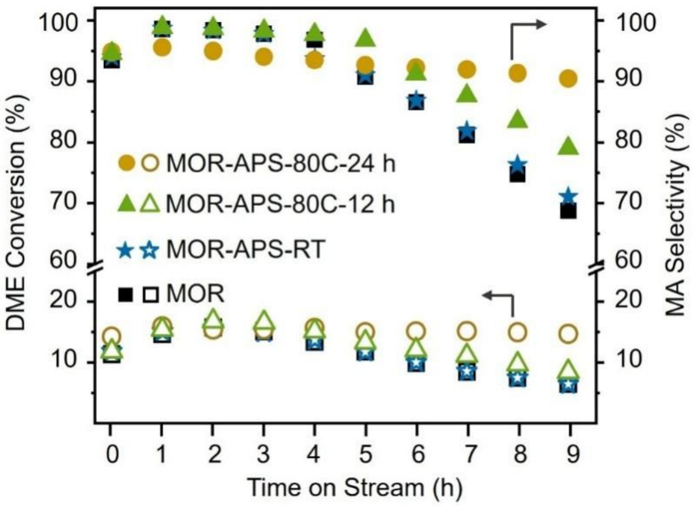

Figure S15. DME conversion and MA selectivity over the parent MOR and MOR catalysts with different persulfate treatment time. Reaction conditions: 200 °C, 2 MPa, DME/CO/N_2=5/35/60, GHSV=3600 mL g^{-1} h^{-1}.

An additional sample (MOR-APS-80C-12h) prepared via 12-hour APS treatment at 80°C, alongside the original 24-hour treated sample (renamed MOR-APS-80C-24h), establishes a controlled gradient of 12-ring Al removal: pristine MOR (1391 μmol/g) → MOR-APS-80C-12h (1142 μmol/g) → MOR-APS-80C-24h (967 μmol/g), while 8-ring Al content remained stable (538–521 μmol/g). Catalytic testing revealed a direct correlation between 12-ring Al reduction and enhanced stability/methyl acetate (MA) selectivity (**Figure S15**). This aligns with prior work[6], where selective 12-ring dealumination suppressed coke formation in larger channels, improving durability without sacrificing activity. These results conclusively demonstrate that the observed performance improvements are directly attributable to targeted 12-ring Al removal, validating the effectiveness of our approach in tailoring zeolite functionality.

Table S1. Crystallographic data of the SXRD measurements of the pristine MOR.

Sample	MOR
Crystal system	orthorhombic
Space group	Cmcm
2θ range for refinement (°)	2-45
Number of independent parameters	47
Number of hkls	1103
Refinement method(s)	Rietveld
a (Å)	18.12988(8)
b (Å)	20.41668(1)
c (Å)	7.49221(1)
V ($Å^3$)	2775.84(5)
R_{wp} / R_p / R_{exp} /R_{Bragg} (%)	6.93/4.94/1.28/4.06
GOF(χ^2)	5.37
Wavelength (Å)	0.700294(6)
2θ zero point(°)	-0.0057(9)
Number of equivalent positions	16

Table S2. Atomic parameters from the Rietveld refinement of the pristine MOR derived from the SXRD measurements.

Species	Atom	x	y	z	SOF[a]	B_{eq}[b] (Å^2)
Zeolite framework	O01	0.2811(5)	0.0000	0.0000	1	2.1394(6)
	O02	0.3268(5)	0.0795(5)	0.2500	1	2.1394(6)
	O03	0.3757(6)	0.0924(7)	0.9243(6)	1	2.1394(6)
	O04	0.2391(5)	0.1223(6)	0.9992(6)	1	2.1394(6)
	O05	0.3253(4)	0.3089(6)	0.2500	1	2.1394(6)
	O06	0.2500	0.2500	0.0000	1	2.1394(6)
	O07	0.3740(5)	0.3039(0)	0.9227(5)	1	2.1394(6)
	O08	0.0000	0.4009(3)	0.2500	1	2.1394(6)
	O09	0.0906(3)	0.3009(5)	0.2500	1	2.1394(6)
	O10	0.0000	0.2044(4)	0.2500	1	2.1394(6)
	Si01	0.3057(3)	0.0736(3)	0.0435(9)	1	0.1535(9)
	Si02	0.3028(3)	0.3106(4)	0.0437(8)	1	0.1535(9)
	Si03	0.0848(2)	0.3791(2)	0.2500	1	0.1535(9)
	Si04	0.0848(2)	0.2227(2)	0.2500	1	0.1535(9)
NH_4^+	N_s1	0.5431(2)	0.7228(1)	0.7487(9)	0.64643	9.0191(1)
	N_s2	0.5424(7)	0.4126(5)	0.4685(7)	0.43354	9.0191(1)
	N_s3	0.6179(7)	0.5005(9)	0.5936(7)	0.93518	9.0191(1)
	N_s4	0.9324(1)	0.6052(2)	0.2495(1)	0.32607	9.0191(1)

[a]B_{eq} refers to the isotropic displacement factor or the thermal parameter.

[b]S_{OF} represents the site occupancy factor per asymmetric unit.

All T (T = Si/Al) of MOR were assigned with the same B_{eq}.

All O of MOR share the same B_{eq}.

Table S3. Crystallographic data of the SXRD measurements of APS-introduced MOR.

Sample	APS-introduced MOR
Crystal system	orthorhombic
Space group	Cmcm
2θ range for refinement (°)	2-45
Number of independent parameters	40
Number of hkls	2274
Refinement method(s)	Rietveld
a (Å)	18.12699(7)
b (Å)	20.40872(6)
c (Å)	7.49862(9)
V (Å^3)	2774.11(6)
R_{wp} / R_p / R_{exp} /R_{Bragg} (%)	7.41/5.44/1.27/4.99
GOF(χ^2)	5.87
Wavelength (Å)	0.700294(6)
2θ zero point(°)	-0.0057(9)
Number of equivalent positions	16

Table S4. Atomic parameters from the Rietveld refinement of the APS-introduced MOR derived from the SXRD measurements.

Species	Atom	x	y	Z	SOF[a]	B_{eq}[b]($Å^2$)
Zeolite framework	O01	0.2811(5)	0.0000	0.0000	1	0.8202(4)
	O02	0.3268(5)	0.0795(5)	0.2500	1	0.8202(4)
	O03	0.3757(6)	0.0924(7)	0.9243(6)	1	0.8202(4)
	O04	0.2391(5)	0.1223(6)	0.9992(6)	1	0.8202(4)
	O05	0.3253(4)	0.3089(6)	0.2500	1	0.8202(4)
	O06	0.2500	0.2500	0.0000	1	0.8202(4)
	O07	0.3740(5)	0.3039(0)	0.9227(5)	1	0.8202(4)
	O08	0.0000	0.4009(3)	0.2500	1	0.8202(4)
	O09	0.0906(3)	0.3009(5)	0.2500	1	0.8202(4)
	O10	0.0000	0.2044(4)	0.2500	1	0.8202(4)
	Si01	0.3057(3)	0.0736(3)	0.0435(9)	1	0.7965(7)
	Si02	0.3028(3)	0.3106(4)	0.0437(8)	1	0.7965(7)
	Si03	0.0848(2)	0.3791(2)	0.2500	1	0.7965(7)
	Si04	0.0848(2)	0.2227(2)	0.2500	1	0.7965(7)
NH_4^+	N_s1	0.5259(6)	0.7461(1)	0.8068(9)	0.75051	9.60174
	N_s2	0.4985(1)	0.4288(8)	0.4813(9)	0.08855	7.03262
	N_s3	0.6077(2)	0.4979(2)	0.5709(3)	0.76401	0.1000
Persulfate	S_4	-0.1168(1)	-0.0256(6)	0.4050(6)	0.02290	0.1000
	S_9	-0.0171(9)	-0.0638(8)	-0.1324(9)	0.02290	0.1000
	O_5	-0.1020(5)	0.0602(7)	0.4245(7)	0.02290	0.1000
	O_6	-0.1990(1)	-0.0347(8)	0.5325(3)	0.02290	0.1000
	O_7	-0.1031(2)	-0.0501(1)	0.1796(2)	0.02290	0.1000
	O_8	-0.0308(8)	-0.0394(5)	0.0929(5)	0.02290	0.1000
	O_3	-0.0434(6)	-0.0684(3)	0.5116(3)	0.02290	0.1000
	O_10	0.0746(3)	-0.0447(8)	-0.1986(7)	0.02290	0.1000
	O_11	-0.0151(8)	-0.1509(6)	-0.1450(5)	0.02290	0.1000

O_12	-0.0682(1)	-0.0112(6)	-0.2761(1)	0.02290	0.1000

[a]B_{eq} refers to the isotropic displacement factor or the thermal parameter.

[b]SOF represents the site occupancy factor per asymmetric unit.

All T (T = Si/Al) of MOR were assigned with the same B_{eq}.

All O of MOR share the same B_{eq}.

Table S5 Quadrupolar parameters of the Al sites in the MOR zeolite and APS-treated MOR samples.

Sample	Quadrupolar parameters[a]	Ta	Tb	Tc	Td
MOR	δ_{iso} (ppm)	58.3	60.5	57.4	56.0
	C_q (MHz)	2.1±0.3	2.3±0.2	1.9±0.3	2.0±0.2
	η	0.5	0.5	0.5	0.5
MOR-APS-80C	δ_{iso} (ppm)	58.3	60.2	57.8	57.1
	C_q (MHz)	2.4±0.2	2.4±0.2	2.2±0.2	2.1±0.1
	η	0.5	0.5	0.5	0.5

[a] Isotropic chemical shifts (δ_{iso}), quadrupole interaction constants (C_q) and asymmetry parameter (η) were extracted from the MQMAS NMR spectra by fitting the representative slices parallel to the F2 dimension at selected F1 chemical shift positions.[6]

References

1. J. Filik, A. Ashton, P. Chang, P. Chater, S. Day, M. Drakopoulos, M. Gerring, M. Hart, O. Magdysyuk, S. Michalik. Processing two-dimensional X-ray diffraction and small-angle scattering data in DAWN 2. *J. Appl. Crystallogr.* **2017**, 50, 959-966.

2. C. Liang, C.-F. Huang, N. Mohanty, R. M. Kurakalva. A rapid spectrophotometric determination of persulfate anion in ISCO. *Chemosphere* **2008**, 73, 1540-1543.

3. T. Chen, B. Huang, S. Day, C. C. Tang, S. C. E. Tsang, K. Y. Wong, T. W. B. Lo. Differential adsorption of l- and d-lysine on achiral MFI zeolites as determined by synchrotron X-ray powder diffraction and thermogravimetric analysis. *Angew. Chem. Int. Ed.* **2020**, 59, 1093-1097.

4. T. Chen, S. J. D., C. C. Tang, T. W. B. Lo, Enantiospecificity in achiral zeolites for asymmetric catalysis. *Phys. Chem. Chem. Phys.* **2020**, 22, 18757-18764.

5. Z. Qin, K. A. Cychosz, G. Melinte, H. El Siblani, J. P. Gilson, M. Thommes, C. Fernandez, S. Mintova, O. Ersen, V. Valtchev. Opening the cages of faujasite-type zeolite. *J. Am. Chem. Soc.* **2017**, 139, 17273-17276.

6. R. Liu, B. Fan, W. Zhang, L. Wang, L. Qi, Y. Wang, S. Xu, Z. Yu, Y. Wei, Z. Liu. Increasing the number of aluminum atoms in T3 sites of a mordenite zeolite by low-pressure $SiCl_4$ treatment to catalyze dimethyl ether carbonylation. *Angew. Chem. Int. Ed.* **2022**, 61, e202116990.

7. Z. Xiong, G. Qi, E. Zhan, Y. Chu, J. Xu, J. Wei, N. Ta, A. Hao, Y. Zhou, F. Deng. Experimental identification of the active sites over a plate-like mordenite for the carbonylation of dimethyl ether. *Chem* **2023**, 9, 76-9.

8. J. Zhang, X. Ding, H. Liu, D. Fan, S. Xu, Y. Wei, Z. Liu. Study on the framework aluminum distributions of HMOR zeolite and identification of active sites for dimethyl ether carbonylation reaction. *Acta Chim. Sinica* **2022**, 80, 590.
